# Machine learning for discovering missing or wrong protein function annotations: A comparison using updated benchmark datasets

**DOI:** 10.1186/s12859-019-3060-6

**Published:** 2019-09-23

**Authors:** Felipe Kenji Nakano, Mathias Lietaert, Celine Vens

**Affiliations:** 1 KU Leuven, Campus KULAK - Department of Public Health and Primary Care, Etienne Sabbelaan 53, Kortrijk, 8500 Belgium; 2 ITEC - imec, Etienne Sabbelaan 51, Kortrijk, 8500 Belgium; 3 Howest University of Applied Sciences, Campus Brugge Station, Rijselstraat 5, Brugge, 8200 Belgium

**Keywords:** Hierarchical multi-label classification, Protein function prediction, Benchmark datasets

## Abstract

**Background:**

A massive amount of proteomic data is generated on a daily basis, nonetheless annotating all sequences is costly and often unfeasible. As a countermeasure, machine learning methods have been used to automatically annotate new protein functions. More specifically, many studies have investigated hierarchical multi-label classification (HMC) methods to predict annotations, using the Functional Catalogue (FunCat) or Gene Ontology (GO) label hierarchies. Most of these studies employed benchmark datasets created more than a decade ago, and thus train their models on outdated information. In this work, we provide an updated version of these datasets. By querying recent versions of FunCat and GO yeast annotations, we provide 24 new datasets in total. We compare four HMC methods, providing baseline results for the new datasets. Furthermore, we also evaluate whether the predictive models are able to discover new or wrong annotations, by training them on the old data and evaluating their results against the most recent information.

**Results:**

The results demonstrated that the method based on predictive clustering trees, Clus-Ensemble, proposed in 2008, achieved superior results compared to more recent methods on the standard evaluation task. For the discovery of new knowledge, Clus-Ensemble performed better when discovering new annotations in the FunCat taxonomy, whereas hierarchical multi-label classification with genetic algorithm (HMC-GA), a method based on genetic algorithms, was overall superior when detecting annotations that were removed. In the GO datasets, Clus-Ensemble once again had the upper hand when discovering new annotations, HMC-GA performed better for detecting removed annotations. However, in this evaluation, there were less significant differences among the methods.

**Conclusions:**

The experiments have showed that protein function prediction is a very challenging task which should be further investigated. We believe that the baseline results associated with the updated datasets provided in this work should be considered as guidelines for future studies, nonetheless the old versions of the datasets should not be disregarded since other tasks in machine learning could benefit from them.

## Background

Due to technological advancements, the generation of proteomic data has increased substantially. However, annotating all sequences is costly and time-consuming, making it often unfeasible [1]. As a countermeasure, recent studies have employed machine learning methods due to their capacities of automatically predicting protein functions.

More specifically, protein function prediction is generally modeled as a hierarchical multi-label classification (HMC) task. HMC is a classification task whose objective is to fit a predictive model *f* which maps a set of instances *X* to a set of hierarchically organized labels *Y*, while respecting hierarchy constraints among *Y* [2, 3]. The hierarchy constraint states that whenever a particular label *y*_*i*_ is predicted, all ancestors labels of *y*_*i*_ up to the root node of the hierarchy must be predicted as well.

In the machine learning literature when proposing a new method, this method is typically compared to a set of competitor methods on benchmark datasets. For HMC, many studies [2–22] utilized the benchmark datasets proposed in [2]. These datasets are available at https://dtai.cs.kuleuven.be/clus/hmcdatasets/ and contain protein sequences from the species *Saccharomyces cerevisiae* (yeast) whose functions are mapped to either the Functional Catalogue (FunCat) [24] or Gene Ontology (GO) [23]. The task associated with these datasets is to predict the functions of a protein, given a a set of descriptive features (e.g., sequence, homology or structural information).

**Fig. 1:**
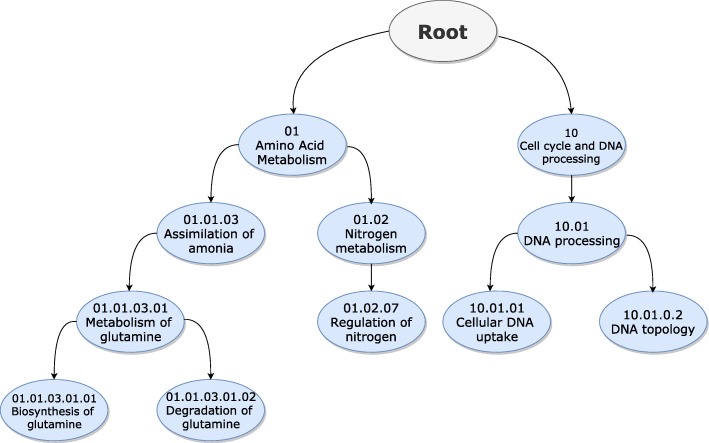
Partial representation of the FunCat. Each node represents a protein function, and each node can only have a single parent node

**Fig. 2:**
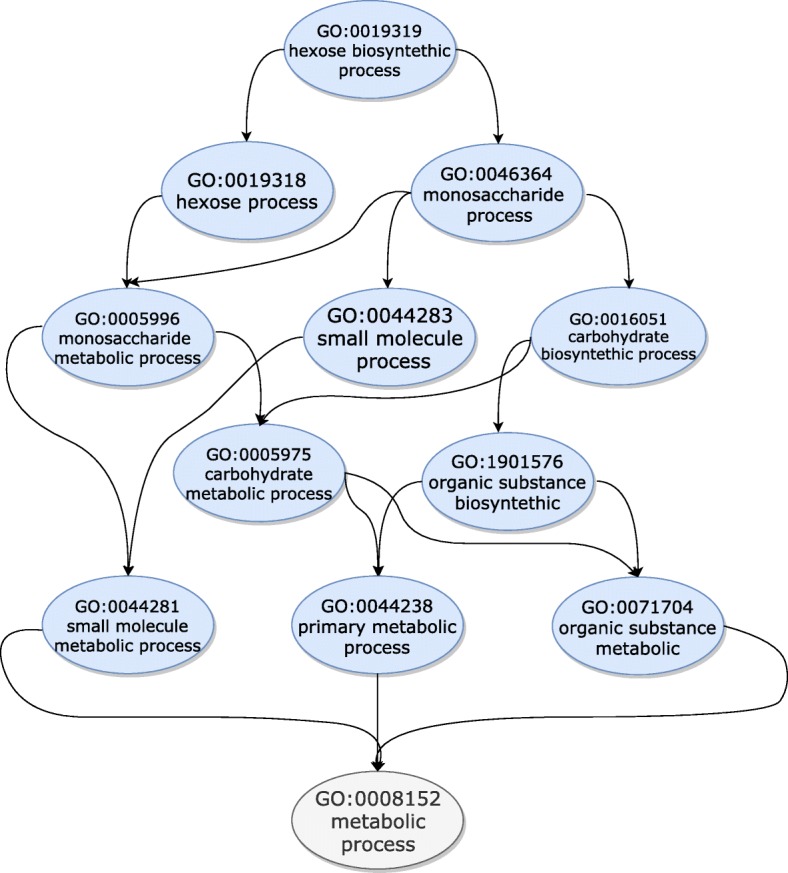
Partial representation of the Gene Ontology. Each node represents a term, and terms can have multiple parent terms

FunCat and GO are different types of hierarchies. In FunCat (Fig. 1), labels are structured as a tree, meaning that they can have only a single parent label [24]. The GO (Fig. 2), however, allows labels to have multiple parent labels, forming a directed acyclic graph [23]. This complicates the fulfillment of the hierarchy constraint, since multiple classification paths are allowed throughout the graph.

**Fig. 3:**
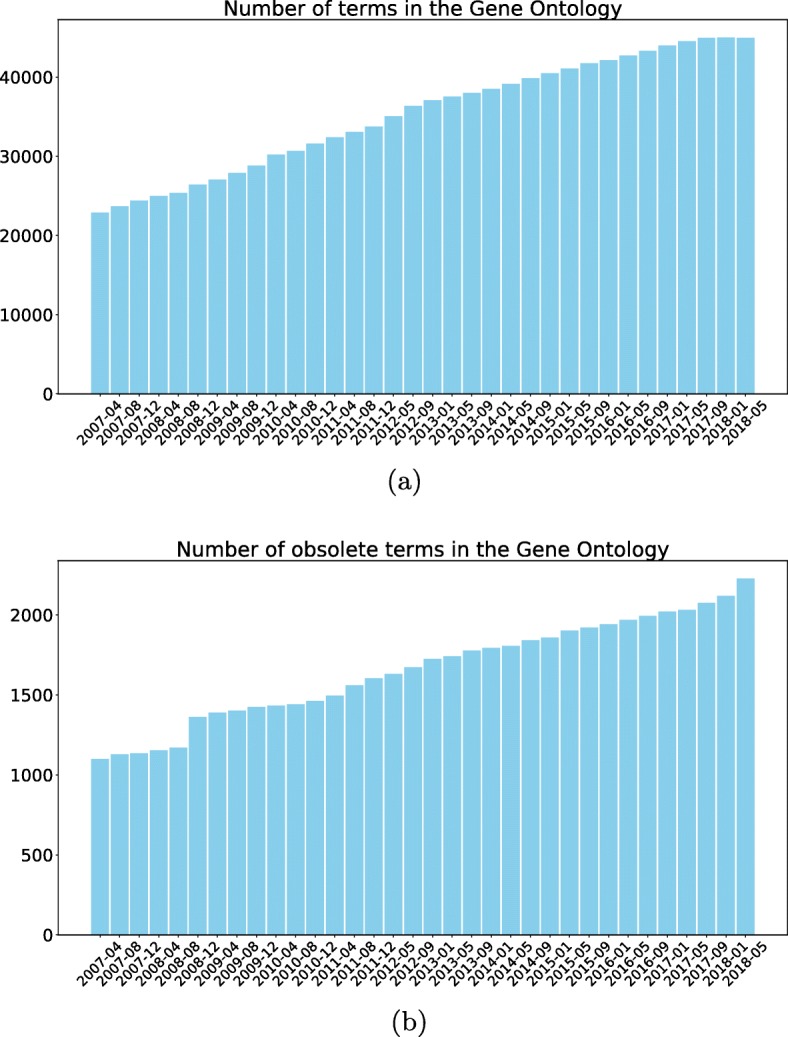
Quantification of terms in the Gene Ontology since 2007. **a** Number of terms in the Gene Ontology. **b** Number of obsolete terms in the Gene Ontology

These benchmark datasets were introduced to the HMC community in 2007, and, thus, the functional labels associated with each protein can be considered outdated. There are two reasons for this. First, functional annotations are updated on a regular basis. Second, as can be seen in Fig. 3a, there was a drastic increase in the number of terms throughout the Gene Ontology since the creation of these datasets (January 2007). A similar observation can be made for the number of obsolete terms as shown in Fig. 3b. Accordingly, one of the main goals of this article is to provide updated versions of these widely used HMC benchmark datasets to the research community.

Using these new datasets, we present a comparison among four recent and open-source HMC methods that can be considered state-of-the-art,thus providing baseline performances as guidelines for future research on this topic. Finally, having two different versions of the same datasets provides us with the unique opportunity to be able to evaluate whether these HMC methods are able to generalize when learning from data with mislabeled instances. In particular, we evaluate whether they were able to predict the correct label in cases where the label has been altered since 2007. In order to do so, we propose an evaluation procedure where a predictive model is trained using the data from 2007, but tested with data from 2018.

The major contributions of this work are the following: i) We provide new benchmark datasets for HMC^1^; ii) We provide baseline results for the new datasets; iii) We provide an evaluation procedure and results that evaluate whether HMC methods are able to discover new or wrong annotations.

The remainder of this article is organized as follows. “Related work” section presents an overview of studies on HMC which have used the functional annotation benchmark datasets proposed in 2007. “Updated datasets” section provides a description on how the datasets were updated, together with a quantification of new labels and annotations. In “Results” section, we present the results of our experiments. In “Discussion” section, we discuss our results. In “Conclusion” section we present our conclusion. Finally, “Methods” section contains the HMC methods employed and the evaluation strategies;

## Related work

In this section, we provide a literature overview of studies that have used the datasets addressed in this work, and a brief review on hierarchical multi-label classification applications. In Table 1, we present studies which have used the FunCat and GO datasets.

**Table 1:** Review on HMC studies which used FunCat and GO datasets

#Year	#Method	#Approach	#Hierarchy
2019	Genetic algorithms [4]	Global	FunCat
2018	Neural networks [5]	Local	FunCat and GO
2018	Neural networks [6]	Global	FunCat and GO
2018	Neural networks [7]	Local	GO
2018	Neural networks and genetic Algorithms [25]	Global	FunCat and GO
2017	Partial least squares [8]	Global	FunCat and GO
2017	Support vector machines [9]	Local	GO
2017	Ant colony optimization [10]	Global	FunCat and GO
2017	K-Nearest Neighbours [26]	Global	GO
2016	Various [11]	Local	FunCat and GO
2016	Neural networks [3]	Local	FunCat
2016	Predictive clustering trees [27]	Global	FunCat and GO
2015	Bayesian optimization [12]	Local	FunCat and GO
2015	Decision trees [13]	Global	FunCat and GO
2015	Neural networks [14]	Local	GO
2014	Genetic algorithm [15]	Global	GO
2014	Naive Bayes [16]	Local	FunCat and GO
2012	Various [28]	Local	FunCat and GO
2013	Centroid based classification [17]	Global	FunCat and GO
2013	Predictive clustering trees [18]	Global	FunCat and GO
2013	Grammatical evolution [29]	Global	FunCat and GO
2012	Genetic algorithms [19]	Global	FunCat
2011	Various [30]	Local	FunCat
2012	Neural network [31]	Global	GO
2011	Bayesian optimization [20]	Local	FunCat and GO
2011	Neural network [30]	Local	FunCat
2011	Predictive clustering trees [21]	Global	FunCat and GO
2010	Artificial ant colony [22]	Global	FunCat and GO
2010	Support vector machines [32]	Local	FunCat
2008	Predictive clustering trees [2]	Global and local	FunCat and GO

In the HMC literature, methods are separated into two approaches: local and global. The difference between these approaches relies on how their predictive models are designed. The local approach employs machine learning decompositions where the task is divided into smaller classification problems, then the solutions of the sub-problems are combined to solve the main task. As an advantage, any predictive model, or even an ensemble of models, can be incorporated into the solution.

According to Silla and Freitas [33], the local approach is further divided into three strategies: Local Classifier per Level [3, 5, 14, 25, 30], Local Classifier per Node [7, 9] and Local Classifier per Parent Node [11, 16]. As their name suggest, these strategies train a predictive model for each level, node or parent node of the hierarchy, respectively. Allowing many types of decomposition is particularly interesting, since different problems may require different solutions. For instance, when handling large hierarchies, the usage of the Local Classifier per Parent Node and Local Classifier per Node result in a large number of classifiers being trained, making the Local Classifier per Level strategy more computationally efficient as it requires only one predictive model per level. However, the hierarchy may contain many labels per level, forcing the models to distinguish among them, and possibly making the task more difficult.

Using several strategies, Cerri and De Carvalho [32] investigated how problem transformation methods from the non-hierarchical multi-label literature, which decompose the task into smaller problems similarly to the local approach, behave on the HMC context using Support Vector Machines. Cerri et al. [3, 14, 30] use the Local Classifier per Level by training one neural network for each level of the hierarchy where prediction probabilities of the previous level are used as extra attributes for the neural network associated to the next level. Wehrmann et al. [5] extended this idea with an extra global loss function, allowing gradients to flow across all neural networks. Li [34] proposed to use this strategy with deep neural networks to predict the commission number of enzymes. In a follow up work, Zou et al. [35] extended this method by enabling the prediction of multi-functional enzymes.

The work of Feng et al. [9] proposed to use the Local Classifier per Node strategy by training one Support Vector Machine for each node of the hierarchy combined with the SMOTE oversampling technique. This work was slightly improved in Feng et al. [7] where the Support Vector Machines were replaced by Multi-Layer Perceptron and a post-prediction method based on Bayesian networks was used. Also using Support Vector Machines, the studies of Bi and Kwok [12, 20] proposed new loss functions specific for HMC which were optimized using Bayes optimization techniques. On a similar manner, Vens et al. [2] proposed to train Predictive Clustering Trees, a variant of decision trees which create splits by minimizing the intra-cluster variance, for each node, and also an alternative version where one predictive model is trained per edge.

Ramirez et al. [11, 16] employed the Local Classifier per Parent Node by training one predictive model per parent node of the hierarchy and augmenting the feature vectors with predictions from ancestors classifiers. On a similar note, Kulmanov et al. [36] proposed to train a predictive model for each sub-ontology of the Gene Ontology, combining features automatically learned from the sequences and features based on protein interactions.

Differently from the local approach, the global one employs a single predictive model which is adapted to handle the hierarchy constraint and relationships among classes. When compared to the local approach, the global one tends to present lower computational complexity, due to the number of models trained. However, its implementation is more complex, since traditional classifiers can not be used straightforwardly. The global approach is further divided into two strategies: algorithm adaptation and rule induction.

As its name suggests, the algorithm adaptation strategy consists of adapting a traditional algorithm to handle hierarchical constraints. Masera and Blanzieri [6] created a neural network whose architecture incorporates the underlying hierarchy, making gradient updates flow from the neurons associated to the leaves up neurons associated to their parent nodes; Sun et al. [8] proposed to use Partial Least Squares to reduce both label and feature dimension, followed by an optimal path selection algorithm; Barros et al. [17] proposed a centroid based method where the training data is initially clustered, then predictions are performed by measuring the distance between the new instance and all clusters, the label set associated to the closest cluster is given as the prediction; Borges and Nievola [31] developed a competitive neural network whose architecture replicates the hierarchy; Vens et al. [2] also proposed to train a single Predictive Clustering Tree for the entire hierarchy; as an extension of [2], Schietgat et al. [21] proposed to use ensemble of Predictive Clustering Trees; Stojanova et al. [18] proposed a slight modification for Predictive Clustering Trees in which the correlation between the proteins is also used to build the tree.

In the rule induction strategy, optimization algorithms are designed to generate classification rules which consist of conjunctions of attribute-value tests, i.e. many *if*→*then* tests connected by the boolean operator ∧. In this regard, several studies from Cerri et al. [4, 15, 19] proposed to use Genetic Algorithms with many different fitness functions. Similarly, other optimization algorithms such as Ant Colony Optimization [10, 22] and Grammar Evolution [29] were also investigated in this context.

Additionally, some studies have also addressed similar topics to HMC. For instance, Cerri et al. [25] examined how Predictive Clustering Trees can be used to perform feature selection using Neural Networks and Genetic Algorithms as base classifiers. Almeida and Borges [26] proposed an adaptation of K-Nearest Neighbours to address quantification learning in HMC. Similarly, Triguero and Vens [27] investigated how different thresholds can increase the performance of Predictive Clustering Trees in this context.

Other application domains have also explored HMC, such as managing IT services [37, 38], text classification on social media [39], large scale document classification [40] and annotation of non-coding RNA [41]. It can even be applied to non-hierarchical multi-label problems where artificial hierarchies are created [42].

## Updated datasets

In this section, we present an overall description of the datasets and their taxonomies, followed by details on how we updated both FunCat and Gene Ontology versions. The resulting updated versions are available at https://www.kuleuven-kulak.be/nl/onderzoek/itec/projects/research-focus/software.

### Overall description

Clare [43] originally proposed 12 datasets containing features extracted from protein sequences of the organism *Saccharomyces cerevisiae* (yeast) whose targets are their protein functions. These 12 datasets contain largely the same proteins, nonetheless differ in their descriptive features. Furthermore, these datasets are divided into train, test and validation sets.

It is known that the yeast and human genomes have many similar genes, furthermore yeast is considerably cheaper and experiment-wise efficient when compared to other species, making it a widely addressed subject in bioinformatics applications [44]. In Table 2, we provide more information about these datasets.

**Table 2:** Statistical information on the 2007 datasets

Dataset	#Features	#Train	#Valid	#Test	#FunCat 2007	#GO 2007
Cellcycle	77	1628	848	1281	499	4122
Church	27	1630	844	1281	499	4122
Derisi	63	1608	842	1275	499	4116
Eisen	79	1058	529	837	461	3570
Expr	551	1639	849	1291	499	4128
Gasch1	173	1634	846	1284	499	4122
Gasch2	52	1639	849	1291	499	4128
Hom	47034	1669	870	1315	499	5828
Pheno	69	656	353	582	455	3124
Seq	478	1701	879	1339	499	4130
Spo	80	1600	837	1266	499	4116
Struc	19628	1665	860	1313	499	5838

The Hom dataset presents information between analogous (similar) yeast genes. Using an homology engine, such as BLASTn 2, other similar yeast genes are discovered. Then, properties between the sequences from the dataset and their analogous ones are measured. The Pheno dataset contains phenotype data based on knock-out mutants. Each gene is removed to form a mutant strain, and the corresponding change in phenotype as compared to the wild type (no mutation) is observed after growing both strains on different growth media. The Seq dataset stores features extracted from the amino acid sequences of the proteins, such as molecular weight, length and amino acid ratios. As its name suggests, the Struc dataset contains features based on the second structure of the proteins annotated in a binary format. In the case of an unknown structure, the software PROF [45] was used to predict it. Known structures were promptly annotated. All the other datasets were constructed based on the expression of genes recorded across an entire genome using microchips [43].

As an extension to these datasets, Vens [2] mapped the targets to the Gene Ontology taxonomy. Additionally, the FunCat annotations used by Clare [43] were updated.

FunCat is an organism independent functional taxonomy of proteins functions which is widely adopted throughout bioinformatics. As shown in Fig. 1, FunCat places generic functions in high levels of the taxonomy, then it sequentially divides such functions into specific ones, forming a tree-shaped hierarchy where each function has one ancestor function. From the machine learning perspective, FunCat is used as an underlying hierarchy of labels. Thus, each protein function is addressed as a label in a classification task where the relationships established by FunCat are taken in account.

Similarly, the Gene Ontology (GO) is a taxonomy whose main goal consists of defining features of genes in an accurate and species independent fashion [23]. More specifically, the GO is composed of three sub-ontologies: molecular function, cellular component and biological process. The molecular function sub-ontology contains information about activities performed by gene products in the molecular-level. The cellular component sub-ontology, as its name suggests, describes the locations where gene products perform functions. Finally, the biological process sub-ontology annotates processes performed by multiple molecular activities.

All information in the GO is described using terms which are nodes with an unique ID, a description and their relationship with other terms. Due to these relationships, the GO is defined as a directed acyclic graph in the machine learning literature, making it a challenging task due to the substantial high number of terms, and many intrinsic relationships among them. Figure 2 presents a small part of the GO.

### FunCat update

**Fig. 4:**
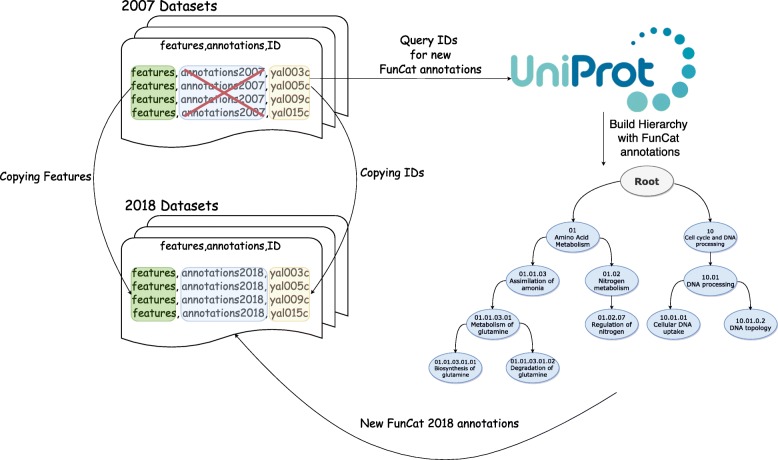
Procedure used to update each FunCat dataset. The sequence IDs are extracted from the 2007 dataset, and used to query new annotations using UniProt. A hierarchy (subset of FunCat) is built using the new annotations. Finally, the old annotations are removed, and the new dataset is created by concatenating the new annotations with the feature vector and IDs

In order to update these datasets, we have performed the procedure described in Fig. 4. Using the IDs from the sequences, we have queried UniProt, obtaining new annotated functions for the sequences. Next, we built the hierarchy of each dataset, and replaced the old annotations by the new ones, i.e. we have removed entirely the annotations from 2007, and concatenated the new annotations with the original features. Mind that each dataset described in Table 2 uses a slightly different FunCat subset. The hierarchies differ between the datasets, because the protein subset differs as seen in Table 2, since not every protein can be found in every original dataset by Clare.

In Table 3, we compared the 2007 datasets with the 2018 versions w.r.t. their label set. There was a significant increase in the number of labels across the hierarchy. More specifically, in the third and fourth level where the mean number of labels has increased from 175 to 208 and 140 to 168 respectively. A smaller increase is also noticeable in the first, second and last level.

**Table 3:** Comparison between the number of labels per level in FunCat 2007 and FunCat 2018

	FunCat 2007	FunCat 2018
Cellcycle	18/80/178/142/77/4	20/86/210/171/92/6
Church	18/80/178/142/77/4	20/86/210/171/92/6
Derisi	18/80/178/142/77/4	20/86/210/171/92/6
Expr	18/80/178/142/77/4	20/86/210/171/92/6
Eisen	18/76/165/131/67/4	19/84/201/159/83/6
Gasch1	18/80/178/142/77/4	20/86/210/171/92/6
Gasch2	18/80/178/142/77/4	20/86/210/171/92/6
Hom	18/80/178/142/77/4	21/86/210/171/92/6
Pheno	18/74/165/129/65/4	20/86/198/156/83/5
Spo	18/80/178/142/77/4	20/86/210/171/92/6
Seq	18/80/178/142/77/4	20/86/210/171/93/6
Struc	18/80/178/142/77/4	20/86/210/171/93/6
Mean	18/79/175/140/75/4	20/85/208/168/90/5

In Table 4, we presented for each dataset the number of instances with annotations per level. In this case, there was a slight increase in deeper levels, whereas the mean number of annotated instances on the second and third level has decreased in all datasets.

**Table 4:** Comparison between the number of annotated instances per level for FunCat 2007 and FunCat 2018

Dataset		Number of instances per level - FunCat 2007	Number of instances per level - FunCat 2018
Cellcycle	Train	1628/1610/1472/975/303/11	1628/1552/1474/1057/370/16
	Test	1281/1272/1163/766/245/8	1281/1222/1159/815/274/15
	Valid	848/836/756/492/164/10	848/804/755/532/192/13
Church	Train	1630/1612/1474/976/302/11	1630/1554/1476/1059/372/16
	Test	1281/1272/1164/764/243/8	1281/1222/1160/813/272/16
	Valid	844/832/752/490/164/10	844/801/752/529/192/13
Derisi	Train	1608/1590/1456/969/300/11	1608/1535/1459/1052/366/16
	Test	1275/1266/1153/761/243/8	1275/1216/1151/812/274/15
	Valid	842/831/751/489/164/10	842/800/752/531/193/13
Expr	Train	1639/1621/1481/979/303/11	1639/1563/1483/1062/372/16
	Test	1291/1282/1173/767/245/8	1291/1231/1168/817/275/16
	Valid	849/837/757/493/164/10	849/805/756/533/192/13
Eisen	Train	1058/1054/997/667/210/8	1058/1019/987/714/251/11
	Test	837/834/784/517/161/4	837/803/774/543/173/9
	Valid	529/525/493/323/104/7	529/510/489/340/117/7
Gasch1	Train	1634/1616/1477/977/303/11	1634/1558/1479/1060/372/16
	Test	1284/1275/1167/764/243/8	1284/1226/1163/814/273/16
	Valid	846/834/754/491/164/10	846/803/754/531/192/13
Gasch2	Train	1639/1621/1481/979/303/11	1639/1563/1483/1062/372/16
	Test	1291/1282/1173/767/245/8	1291/1231/1168/817/275/16
	Valid	849/837/757/493/164/10	849/805/756/533/192/13
Hom	Train	1669/1607/1470/979/302/11	1669/1548/1470/1059/372/16
	Test	1315/1275/1167/766/245/8	1315/1226/1163/817/275/16
	Valid	870/833/753/492/164/10	870/802/753/532/193/13
Pheno	Train	656/649/593/403/133/6	656/625/590/438/158/8
	Test	582/578/524/348/108/3	582/556/525/372/121/8
	Valid	353/349/316/204/69/6	353/338/311/223/84/5
Spo	Train	1600/1582/1448/963/299/11	1600/1527/1451/1043/365/16
	Test	1266/1257/1146/758/243/8	1266/1208/1144/808/272/15
	Valid	837/826/747/486/163/10	837/795/747/526/191/13
Seq	Train	1701/1639/1499/990/305/11	1701/1578/1497/1072/376/16
	Test	1339/1298/1188/774/248/8	1339/1246/1182/825/278/16
	Valid	879/842/762/497/164/10	879/810/761/538/194/13
Struc	Train	1665/1634/1495/988/304/11	1665/1575/1494/1071/375/16
	Test	1313/1292/1182/770/245/8	1313/1241/1177/821/275/16
	Valid	860/840/760/495/162/10	860/808/759/536/192/13
Mean	Train	1510/1486/1361/903/280/10	1510/1433/1361/979/343/14
	Test	1196/1181/1082/710/226/7	1196/1135/1077/756/253/14
	Valid	783/768/696/453/150/9	783/740/695/490/177/11

Further, we compared the number of annotations per level between the versions from 2007 and 2018 in Table 5. There was a considerable increase in the number of annotations across all levels of the hierarchy. The last level seemed remarkable, as its number of annotations is significantly low in both versions.

**Table 5:** Comparison between the number of annotations per level in FunCat 2007 and FunCat 2018

Dataset		Annotations per Level - 2007	Annotations per Level - 2018
Cellcycle	Train	3915/4629/3553/1727/363/11	4720/6219/4917/2266/536/16
	Test	3162/3744/2865/1351/291/8	3756/4949/3936/1734/399/15
	Valid	2029/2373/1813/865/194/10	2497/3286/2585/1169/283/13
Church	Train	3913/4628/3554/1726/362/11	4723/6225/4923/2269/538/16
	Test	3156/3735/2858/1347/289/8	3754/4948/3928/1729/397/16
	Valid	2021/2362/1805/862/194/10	2486/3267/2572/1164/283/13
Derisi	Train	3883/4586/3537/1716/361/11	4677/6159/4876/2248/529/16
	Test	3157/3741/2849/1343/289/8	3755/4963/3934/1728/399/15
	Valid	2021/2364/1805/858/194/10	2486/3271/2579/1162/283/13
Expr	Train	3931/4646/3568/1732/363/11	4741/6246/4937/2274/538/16
	Test	3179/3762/2879/1354/291/8	3779/4980/3953/1739/400/16
	Valid	2030/2375/1815/866/194/10	2501/3291/2588/1170/283/13
Eisen	Train	2627/3157/2489/1199/259/8	3216/4345/3457/1578/386/11
	Test	2130/2537/1977/929/195/4	2561/3428/2721/1179/269/9
	Valid	1311/1554/1209/584/126/7	1593/2107/1645/732/161/7
Gasch1	Train	3921/4636/3560/1728/363/11	4730/6232/4928/2270/538/16
	Test	3164/3745/2863/1347/289/8	3764/4965/3938/1732/398/16
	Valid	2025/2367/1807/863/194/10	2491/3273/2576/1166/283/13
Gasch2	Train	3931/4646/3568/1732/363/11	4741/6246/4937/2274/538/16
	Test	3179/3762/2879/1354/291/8	3779/4980/3953/1739/400/16
	Valid	2030/2375/1815/866/194/10	2501/3291/2588/1170/283/13
Hom	Train	3971/4649/3566/1733/362/11	4772/6238/4936/2272/538/16
	Test	3196/3742/2866/1355/291/8	3808/4981/3957/1742/400/16
	Valid	2051/2366/1810/864/194/10	2516/3271/2577/1168/284/13
Pheno	Train	1668/1977/1508/709/154/6	2008/2644/2095/939/234/8
	Test	1506/1764/1308/618/129/3	1750/2308/1814/803/177/8
	Valid	881/1014/753/362/81/6	1087/1423/1131/511/133/5
Spo	Train	3854/4551/3512/1705/359/11	4645/6116/4842/2231/527/16
	Test	3133/3709/2831/1339/289/8	3727/4914/3904/1722/397/15
	Valid	2006/2346/1794/855/193/10	2464/3237/2552/1153/281/13
Seq	Train	4037/4731/3626/1754/365/11	4847/6337/5006/2298/544/16
	Test	3242/3800/2914/1368/294/8	3856/5043/4003/1755/403/16
	Valid	2068/2390/1828/872/194/10	2546/3317/2608/1179/285/13
Struc	Train	3994/4718/3615/1750/364/11	4806/6329/4997/2295/543/16
	Test	3207/3779/2890/1360/290/8	3819/5016/3978/1746/399/16
	Valid	2037/2373/1816/865/191/10	2515/3300/2596/1170/282/13
Mean	Train	3637/4296/3304/1600/336/10	4385/5778/4570/2101/499/14
	Test	2950/3485/2664/1255/269/7	3509/4622/3668/1612/369/14
	Valid	1927/2250/1718/819/182/9	2306/3027/2383/1076/260/11

When analyzing the number of annotations that were added and removed in Table 6, the second level presented a higher average number of new annotations despite having fewer annotated instances now. Noticeable increases were also noticed in the third and fourth level.

**Table 6:** Comparison between added and removed annotations in FunCat 2007 and FunCat 2018 per level

Dataset		Number of annotations added	Number of annotations removed
Cellcycle	Train	1083/1875/1585/654/199/6	278/285/221/115/26/1
	Test	794/1414/1231/475/137/8	200/209/160/92/29/1
	Valid	585/1031/873/359/104/4	117/118/101/55/15/1
Church	Train	1087/1881/1589/657/201/6	277/284/220/114/25/1
	Test	802/1431/1234/475/137/9	204/218/164/93/29/1
	Valid	581/1022/867/357/104/4	116/117/100/55/15/1
Derisi	Train	1060/1841/1552/644/192/6	266/268/213/112/24/1
	Test	799/1431/1246/477/139/8	201/209/161/92/29/1
	Valid	581/1024/874/359/104/4	116/117/100/55/15/1
Expr	Train	1088/1885/1590/657/201/6	278/285/221/115/26/1
	Test	801/1428/1235/477/138/9	201/210/161/92/29/1
	Valid	588/1034/874/359/104/4	117/118/101/55/15/1
Eisen	Train	756/1369/1108/458/144/4	167/181/140/79/17/1
	Test	562/1028/856/318/97/6	131/137/112/68/23/1
	Valid	353/632/503/190/47/1	71/79/67/42/12/1
Gasch1	Train	1087/1881/1589/657/201/6	278/285/221/115/26/1
	Test	799/1428/1235/477/138/9	199/208/160/92/29/1
	Valid	582/1023/869/358/104/4	116/117/100/55/15/1
Gasch2	Train	1088/1885/1590/657/201/6	278/285/221/115/26/1
	Test	801/1428/1235/477/138/9	201/210/161/92/29/1
	Valid	588/1034/874/359/104/4	117/118/101/55/15/1
Hom	Train	1130/1885/1600/656/201/6	329/296/230/117/25/1
	Test	842/1447/1251/479/138/9	230/208/160/92/29/1
	Valid	230/208/160/92/29/1	142/117/100/55/15/1
Pheno	Train	450/780/680/274/90/3	110/113/93/44/10/1
	Test	345/642/572/215/54/5	101/98/66/30/6/0
	Valid	253/448/419/171/56/0	47/39/41/22/4/1
Spo	Train	1056/1833/1543/638/192/6	265/268/213/112/24/1
	Test	793/1413/1233/475/137/8	199/208/160/92/29/1
	Valid	573/1008/858/353/103/4	115/117/100/55/15/1
Seq	Train	1145/1909/1616/664/205/6	335/303/236/120/26/1
	Test	849/1456/1252/479/138/9	235/213/163/92/29/1
	Valid	621/1045/881/362/106/4	143/118/101/55/15/1
Struc	Train	1112/1909/1614/664/205/6	300/298/232/119/26/1
	Test	826/1449/1250/478/138/9	214/212/162/92/29/1
	Valid	604/1045/881/360/106/4	126/118/101/55/15/1
Mean	Train	1011/1744/1471/606/186/5	263/262/205/106/23/1
	Test	751/1332/1152/441/127/8	193/195/149/84/26/0
	Valid	511/879/744/306/89/3	111/107/92/51/13/1

### Gene ontology update

**Fig. 5:**
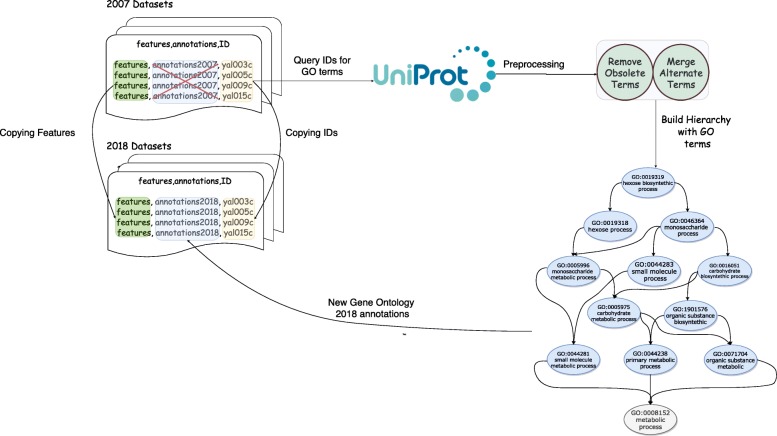
Procedure used to update each Gene Ontology dataset. The sequence IDs are extracted from the 2007 dataset, and used to query new terms using UniProt. Obsolete and replaced terms are removed and merged into a single term, respectively. A hierarchy (subset of the Gene Ontology) is built using the new annotations. Finally, the old annotations are removed, and the new dataset is created by concatenating the new annotations with the feature vector and IDs

In order to update these datasets, we have performed the procedure shown in Fig. 5.

Initially, we queried Universal Protein (UniProt) using the IDs from the protein sequences using their web service^3^, obtaining the GO terms associated to each sequence. Next, we preprocessed the queried terms. The GO keeps track of alternate (secondary) IDs which are different labels with identical meaning, hence we have merged them into a single label. Similarly, we have also removed obsolete annotations since they are deprecated and should not be used anymore. Finally, the old annotations were entirely removed, and the new ones were concatenated to the feature vector. Recall that we are not considering the first level of the Gene Ontology, since it contains 3 root terms which are present in all instances. Further, as for FunCat, each dataset contains only a subset of the entire Gene Ontology.

Mind that since the GO is a directed acyclic graph, annotations can belong to multiple levels. In order to present statistics about these datasets, we are considering the deepest path to determine the level for all labels in Tables 7, 8, 9 10.

**Table 7:** Comparison between the number of labels per level in Gene Ontology 2007 and Gene Ontology 2018

	#1	#2	#3	#4	#5	#6	#7	#8	#9	#10	#11	#12	#13	#14	#15
Cellcycle	33	155	394	597	929	779	631	335	171	63	21	5	9		
	42	223	482	826	1454	1267	1153	870	654	451	324	216	86	13	1
Church	33	155	394	597	929	779	631	335	171	63	21	5	9		
	42	223	481	825	1454	1265	1153	870	654	451	324	216	86	13	1
Derisi	33	155	394	596	927	778	630	334	171	63	21	5	9		
	42	223	481	823	1449	1262	1151	866	651	449	323	216	86	13	1
Expr	33	155	394	599	932	780	631	335	171	63	21	5	9		
	42	223	482	827	1454	1267	1154	870	654	451	324	216	86	13	1
Eisen	33	149	360	524	786	679	539	271	141	55	19	5	9		
	42	217	452	732	1215	1088	975	754	567	388	283	186	78	12	1
Gasch1	33	155	394	597	929	779	631	335	171	63	21	5	9		
	42	223	482	826	1454	1266	1153	870	654	451	324	216	86	13	1
Gasch2	33	155	394	599	932	780	631	335	171	63	21	5	9		
	42	223	482	827	1454	1267	1154	870	654	451	324	216	86	13	1
Hom	33	155	394	597	929	778	633	335	171	63	21	5	9		
	42	223	482	826	1450	1264	1152	868	655	452	323	214	86	13	1
Pheno	33	145	332	489	670	568	460	236	114	49	18	4	6		
	42	192	423	684	1103	999	878	671	494	346	247	150	69	11	1
Spo	33	155	394	596	927	778	630	334	171	63	21	5	9		
	42	223	481	823	1449	1262	1151	867	651	449	323	216	86	13	1
Seq	33	155	394	599	932	780	633	335	171	63	21	5	9		
	42	223	482	828	1456	1269	1154	870	656	452	324	216	86	13	1
Struc	33	155	394	599	932	779	633	335	171	63	21	5	9		
	42	223	482	828	1456	1267	1154	868	655	452	324	216	86	13	1
Mean	33	153	386	582	896	753	609	321	163	61	20	4	8		
	42	219	474	806	1399	1228	1115	834	633	436	314	207	83	12	1

**Table 8:** Comparison between the number of annotated instances per level Gene Ontology 2007 and Gene Ontology 2018

Dataset		Number of instances per level - GO 2007	Number of instances per level - GO 2018
Cellcycle	Train	1625/1598/1598/1591/1571/1531/1307/1072/655/407/193/74/18/10/0	1620/1620/1619/1607/1588/1543/1474/1321/1040/808/539/288/110/17/0
	Test	1278/1258/1258/1255/1242/1213/1047/858/499/319/157/62/13/7/0	1270/1270/1268/1264/1254/1224/1171/1037/800/620/423/252/91/11/3
	Valid	848/824/824/822/812/794/671/537/318/188/93/37/7/4/0	844/843/838/832/825/793/753/663/514/401/267/143/59/10/1
Church	Train	1627/1600/1600/1593/1573/1534/1307/1071/654/406/193/74/18/10/0	1622/1622/1621/1609/1590/1546/1477/1322/1042/809/541/288/110/17/0
	Test	1278/1258/1258/1254/1241/1212/1046/858/501/319/157/62/13/7/0	1269/1269/1267/1263/1253/1224/1171/1037/799/621/424/253/91/11/3
	Valid	844/820/820/818/808/791/669/535/317/187/92/36/6/3/0	840/839/834/828/821/789/749/659/512/399/265/142/58/9/1
Derisi	Train	1594/1567/1567/1560/1540/1501/1282/1058/647/402/191/74/18/10/0	1589/1589/1588/1576/1558/1515/1449/1299/1028/797/532/285/110/17/0
	Test	1257/1237/1237/1234/1222/1193/1029/846/496/317/155/61/13/7/0	1249/1249/1247/1243/1233/1204/1152/1019/792/614/418/252/91/11/3
	Valid	836/812/812/810/802/786/664/533/316/188/93/37/7/3/0	832/831/826/820/813/781/743/656/511/400/268/143/58/9/1
Expr	Train	1636/1609/1609/1602/1582/1542/1313/1075/656/407/193/74/18/10/0	1631/1631/1630/1618/1598/1553/1483/1328/1045/812/541/288/110/17/0
	Test	1288/1268/1268/1264/1251/1222/1054/864/502/319/157/62/13/7/0	1280/1280/1278/1274/1264/1234/1181/1046/805/625/426/253/91/11/3
	Valid	849/825/825/823/813/795/672/538/319/189/93/37/7/4/0	845/844/839/833/826/794/754/664/515/402/268/143/59/10/1
Eisen	Train	1055/1054/1054/1053/1050/1033/909/768/478/304/144/59/16/10/0	1053/1053/1053/1051/1043/1026/995/921/740/582/399/213/78/16/0
	Test	835/834/834/834/834/819/738/612/358/227/117/44/11/7/0	831/831/831/830/826/817/791/712/557/438/299/177/60/10/3
	Valid	528/527/527/526/524/519/453/373/231/141/64/30/4/2/0	526/526/526/526/522/511/496/450/350/279/189/103/44/7/1
Gasch1	Train	1631/1604/1604/1597/1577/1537/1310/1073/655/406/193/74/18/10/0	1626/1626/1625/1613/1593/1549/1480/1325/1043/810/541/288/110/17/0
	Test	1281/1261/1261/1257/1244/1215/1048/859/501/319/157/62/13/7/0	1273/1273/1271/1267/1257/1228/1175/1041/801/623/425/253/91/11/3
	Valid	846/822/822/820/810/793/670/536/317/187/92/36/6/3/0	842/841/836/830/823/791/751/661/513/400/266/142/58/9/1
Gasch2	Train	1636/1609/1609/1602/1582/1542/1313/1075/656/407/193/74/18/10/0	1631/1631/1630/1618/1598/1553/1483/1328/1045/812/541/288/110/17/0
	Test	1288/1268/1268/1264/1251/1222/1054/864/502/319/157/62/13/7/0	1280/1280/1278/1274/1264/1234/1181/1046/805/625/426/253/91/11/3
	Valid	849/825/825/823/813/795/672/538/319/189/93/37/7/4/0	845/844/839/833/826/794/754/664/515/402/268/143/59/10/1
Hom	Train	1660/1633/1633/1626/1606/1567/1306/1068/653/408/196/74/18/10/0	1656/1656/1655/1643/1625/1563/1495/1342/1044/811/543/289/111/17/0
	Test	1309/1289/1289/1285/1273/1245/1050/860/504/320/157/62/13/7/0	1301/1301/1299/1295/1285/1243/1191/1059/804/626/425/252/91/11/3
	Valid	867/843/843/841/831/815/670/536/318/188/92/36/6/3/0	863/862/857/851/844/800/761/672/515/402/268/142/58/9/1
Pheno	Train	653/639/639/638/629/612/511/419/257/160/73/25/8/6/0	650/650/650/643/638/620/587/516/404/322/232/129/55/11/0
	Test	581/573/573/572/564/548/462/383/230/139/73/33/9/5/0	576/576/575/573/569/555/535/474/368/283/207/127/50/6/1
	Valid	352/344/344/343/340/334/277/221/139/82/43/15/2/1/0	350/350/347/346/342/327/312/272/219/170/116/68/34/4/1
Dataset		Number of instances per level - GO 2007	Number of instances per level - GO 2018
Spo	Train	1596/1569/1569/1562/1542/1503/1283/1058/647/402/191/74/18/10/0	1591/1591/1590/1578/1560/1517/1451/1301/1028/797/532/285/110/17/0
	Test	1258/1238/1238/1235/1223/1194/1030/847/495/316/155/61/13/7/0	1250/1250/1248/1244/1234/1205/1153/1019/793/615/419/252/91/11/3
	Valid	836/812/812/810/802/786/664/532/315/187/92/37/7/3/0	832/831/826/820/813/781/743/655/510/399/267/143/58/9/1
Seq	Train	1691/1664/1664/1657/1637/1597/1326/1085/663/413/199/74/18/10/0	1686/1686/1685/1673/1653/1590/1520/1367/1057/823/548/294/111/17/0
	Test	1332/1311/1311/1307/1294/1265/1066/872/506/322/158/62/13/7/0	1324/1324/1322/1318/1308/1263/1209/1074/814/634/430/254/91/11/3
	Valid	836/812/812/810/802/786/664/532/315/187/92/37/7/3/0	832/831/826/820/813/781/743/655/510/399/267/143/58/9/1
Struc	Train	1658/1631/1631/1624/1604/1564/1323/1082/661/411/198/74/18/10/0	1653/1653/1652/1640/1620/1571/1502/1348/1054/820/546/293/111/17/0
	Test	1306/1285/1285/1281/1268/1239/1061/867/503/320/157/62/13/7/0	1298/1298/1296/1292/1282/1246/1192/1058/810/631/428/253/91/11/3
	Valid	859/835/835/833/823/805/674/539/321/191/94/37/7/4/0	855/854/849/843/836/800/759/671/518/405/271/143/59/10/1
Mean	Train	1505/1481/1481/1475/1457/1421/1207/992/606/377/179/68/17/9/0	1500/1500/1499/1489/1472/1428/1366/1226/964/750/502/269/103/16/0
	Test	1268/1268/1265/1252/1223/1047/861/502/316/157/62/13/7/0/0	1278/1278/1276/1272/1261/1228/1176/1042/799/621/425/253/92/11/3s
	Valid	823/823/821/813/797/669/534/315/188/91/36/6/3/0/0	823/822/817/812/805/770/734/649/502/392/263/139/58/9/1

**Table 9:** Comparison between the number of annotations per level in Gene Ontology 2007 and Gene Ontology 2018

		Number of annotations per level - 2007	Number of annotations per level - 2018
Cellcycle	Train	8503/10785/10322/7639/6096/3726/2390/1095/553/243/90/24/13/0/0	10453/18179/19093/17826/14561/9491/6155/4503/2565/1532/896/398/110/11/0
	Test	6933/8801/8476/6284/5030/3040/1964/887/447/200/81/19/7/0/0	9663/15948/16855/15546/12833/8367/5442/3827/2139/1256/771/397/124/12/3
	Valid	4350/5518/5277/3889/3097/1773/1145/536/270/123/52/10/5/0/0	6340/10347/10877/9898/8003/5108/3353/2396/1329/830/488/214/82/8/1
Church	Train	8508/10794/10331/7645/6102/3727/2387/1091/552/243/90/24/13/0/0	12015/20133/20979/19244/15615/10166/6526/4756/2691/1592/931/412/118/12/0
	Test	6935/8798/8469/6279/5025/3035/1963/889/447/200/81/19/7/0/0	9596/15900/16911/15534/12676/8274/5347/3798/2119/1247/755/382/119/13/3
	Valid	4332/5492/5253/3869/3088/1765/1139/534/268/121/50/8/4/0/0	6262/10344/10907/9891/7935/5038/3327/2440/1368/847/485/212/81/8/1
Derisi	Train	8411/10679/10219/7565/6043/3689/2373/1083/549/242/90/24/13/0/0	11873/19951/20719/18965/15482/10070/6528/4684/2655/1566/907/402/116/11/0
	Test	6889/8755/8464/6274/5016/3034/1965/890/448/200/81/19/7/0/0	9622/15942/16865/15572/12852/8389/5465/3837/2149/1262/779/400/124/12/3
	Valid	4335/5497/5263/3882/3092/1766/1141/541/272/123/51/9/4/0/0	6289/10283/10850/9875/7982/5096/3336/2406/1336/833/487/213/80/8/1
Expr	Train	8552/10839/10373/7676/6127/3739/2393/1096/553/243/90/24/13/0/0	12078/20305/21111/19395/15764/10236/6605/4764/2718/1586/922/413/117/11/0
	Test	6990/8872/8532/6325/5062/3052/1972/890/447/200/81/19/7/0/0	9736/16109/17120/15796/12950/8452/5487/3870/2178/1293/788/405/133/16/4
	Valid	4356/5523/5282/3892/3100/1778/1146/537/271/123/52/10/5/0/0	6306/10359/10892/9928/8062/5161/3360/2414/1349/854/514/228/88/8/1
Eisen	Train	5999/7650/7313/5409/4408/2758/1771/830/418/184/72/22/13/0/0	8017/13544/14278/13196/10820/7090/4607/3381/1923/1173/696/310/86/11/0
	Test	4856/6203/5969/4457/3579/2210/1452/637/320/148/56/17/7/0/0	6418/10714/11509/10701/8787/5791/3768/2709/1517/898/529/273/91/11/3
	Valid	2980/3797/3601/2663/2145/1258/807/387/198/89/40/5/3/0/0	4085/6748/7181/6585/5442/3548/2290/1663/924/582/348/161/69/8/1
Gasch1	Train	8528/10814/10349/7658/6113/3732/2389/1092/552/243/90/24/13/0/0	12061/20214/21005/19233/15663/10208/6599/4733/2684/1582/908/403/116/11/0
	Test	6956/8822/8487/6292/5035/3038/1964/889/447/200/81/19/7/0/0	8518/13333/14027/12536/10243/6619/4280/2961/1644/950/554/275/91/11/3
	Valid	4341/5504/5265/3877/3092/1768/1140/534/268/121/50/8/4/0/0	6329/10336/10864/9887/7997/5102/3346/2394/1327/828/486/211/80/8/1
Gasch2	Train	8552/10839/10373/7676/6127/3739/2393/1096/553/243/90/24/13/0/0	12090/20284/21121/19491/15879/10398/6728/4832/2737/1617/935/423/123/14/0
	Test	6990/8872/8532/6325/5062/3052/1972/890/447/200/81/19/7/0/0	9708/16004/16957/15616/12782/8394/5459/3840/2134/1254/761/387/126/13/4
	Valid	4356/5523/5282/3892/3100/1778/1146/537/271/123/52/10/5/0/0	6306/10265/10830/9806/7974/5125/3389/2419/1346/850/494/214/83/8/1
Hom	Train	8677/10965/10503/7722/6213/3749/2391/1092/554/246/90/24/13/0/0	12265/20926/21585/19562/16060/10447/6758/4809/2692/1582/904/399/117/11/0
	Test	7084/8963/8615/6349/5108/3036/1963/892/448/200/81/19/7/0/0	9898/16603/17421/15930/13196/8588/5593/3917/2137/1259/770/397/124/12/3
	Valid	4434/5605/5355/3913/3140/1765/1140/535/269/121/50/8/4/0/0	6461/10747/11197/10075/8198/5214/3427/2436/1326/827/486/211/80/8/1
Pheno	Train	3407/4377/4224/3129/2452/1519/973/417/215/89/32/11/8/0/0	4876/8133/8531/7911/6424/4184/2662/1905/1087/661/407/192/59/6/0
	Test	3127/4000/3899/2915/2267/1398/942/428/207/101/45/13/5/0/0	4414/7363/7818/7229/5914/3902/2564/1869/1050/603/365/193/60/5/1
	Valid	1856/2357/2301/1687/1325/757/510/250/115/54/21/2/2/0/0	2653/4454/4708/4344/3544/2296/1506/1103/629/380/237/113/51/7/1
		Number of annotations per level - 2007	Number of annotations per level - 2018
Spo	Train	8361/10613/10158/7520/6008/3668/2359/1080/548/241/90/24/13/0/0	11956/20176/21045/19222/15723/10209/6545/4692/2649/1572/903/403/116/11/0
	Test	6842/8690/8389/6219/4977/3011/1947/883/446/200/81/19/7/0/0	9570/15835/16752/15450/12760/8325/5423/3809/2135/1255/771/397/124/12/3
	Valid	4307/5460/5226/3855/3072/1753/1133/535/268/122/51/9/4/0/0	6265/10235/10785/9826/7956/5074/3328/2386/1327/829/487/212/80/8/1
Seq	Train	8840/11168/10688/7849/6318/3806/2424/1108/559/249/90/24/13/0/0	12492/21255/21931/19863/16293/10600/6845/4875/2722/1599/908/404/117/11/0
	Test	7203/9111/8747/6441/5189/3087/1985/895/450/201/81/19/7/0/0	10059/16832/17661/16126/13350/8687/5642/3965/2168/1270/774/398/124/12/3
	Valid	4478/5658/5405/3951/3171/1791/1155/542/274/124/52/10/5/0/0	6524/10820/11270/10142/8244/5251/3449/2447/1332/829/488/214/82/8/1
Struc	Train	8688/10998/10540/7777/6238/3797/2418/1106/558/249/90/24/13/0/0	12275/20674/21445/19540/15965/10402/6723/4807/2717/1595/905/403/117/11/0
	Test	7071/8953/8603/6362/5105/3066/1973/892/447/200/81/19/7/0/0	9827/16300/17185/15794/12960/8453/5475/3872/2141/1256/766/398/128/12/3
	Valid	4397/5565/5318/3903/3125/1783/1150/541/273/124/52/10/5/0/0	6409/10520/11013/9977/8079/5156/3388/2418/1332/829/488/214/82/8/1
Mean	Train	7788/9878/9462/6992/5598/3413/2186/998/504/222/82/22/12/0/0	11037/18647/19403/17787/14520/9458/6106/4395/2486/1471/851/380/109/10/0
	Test	6489/8236/7931/5876/4704/2838/1838/830/416/187/75/18/6/0/0	8919/14740/15590/14319/11775/7686/4995/3522/1959/1150/698/358/114/11/3
	Valid	4043/5124/4902/3606/2878/1644/1062/500/251/114/47/8/4/0/0	5852/9621/10114/9186/7451/4764/3124/2243/1243/776/457/201/78/7/1

**Table 10:** Comparison between the number of annotations added and removed in Gene Ontology 2007 and Gene Ontology 2018 per level

		Number of Annotations Added per Level	Number of Annotations Removed per Level
Cellcycle	Train	4586/12181/13927/13852/11608/7784/5025/3959/2284/1408/838/382/103/11/0	1066/2803/3310/2310/2077/1333/834/343/166/76/23/3/0/0/0
	Test	3649/9613/11237/11200/9547/6463/4258/3243/1852/1116/710/379/117/12/3	919/2466/2858/1938/1744/1136/780/303/160/60/20/1/0/0/0
	Valid	2504/6312/7308/7210/5948/3964/2628/2027/1135/746/449/204/78/8/1	514/1483/1708/1201/1042/629/420/167/76/39/13/0/1/0/0
Church	Train	4558/12124/13910/13866/11568/7772/4976/3995/2299/1422/863/390/105/12/0	1051/2785/3262/2267/2055/1333/837/330/160/73/22/2/0/0/0
	Test	3551/9545/11259/11159/9365/6332/4146/3202/1829/1104/693/364/112/13/3	890/2443/2817/1904/1714/1093/762/293/157/57/19/1/0/0/0
	Valid	2447/6328/7340/7201/5885/3901/2599/2070/1178/765/448/204/78/8/1	517/1476/1686/1179/1038/628/411/164/78/39/13/0/1/0/0
Derisi	Train	4516/12053/13780/13684/11491/7708/4986/3933/2270/1401/840/381/103/11/0	1054/2781/3280/2284/2052/1327/831/332/164/77/23/3/0/0/0
	Test	3651/9645/11263/11235/9570/6484/4279/3252/1863/1122/718/382/117/12/3	918/2458/2862/1937/1734/1129/779/305/162/60/20/1/0/0/0
	Valid	2479/6289/7291/7183/5938/3959/2625/2029/1141/749/451/204/77/8/1	512/1478/1701/1190/1036/628/420/169/77/39/13/0/1/0/0
Expr	Train	4603/12271/14055/14010/11713/7843/5050/4009/2333/1420/855/392/104/11/0	1077/2805/3317/2291/2076/1346/838/341/168/77/23/3/0/0/0
	Test	3644/9709/11459/11414/9640/6535/4299/3293/1895/1152/726/387/126/16/4	898/2472/2871/1943/1752/1135/784/313/164/59/19/1/0/0/0
	Valid	2475/6339/7314/7226/6010/4012/2644/2041/1155/770/477/218/84/8/1	525/1503/1704/1190/1048/629/430/164/77/39/15/0/1/0/0
Eisen	Train	2760/7871/9258/9380/7858/5322/3452/2795/1626/1045/639/290/73/11/0	742/1977/2293/1593/1446/990/616/244/121/56/15/2/0/0/0
	Test	2190/6234/7476/7556/6389/4376/2887/2285/1317/789/483/256/84/11/3	628/1723/1936/1312/1181/795/571/213/120/39/10/0/0/0/0
	Valid	1463/3985/4718/4729/4016/2724/1786/1395/776/524/322/156/67/8/1	358/1034/1138/807/719/434/303/119/50/31/14/0/1/0/0
Gasch1	Train	4599/12213/13965/13878/11626/7805/5040/3978/2296/1415/841/382/103/11/0	1066/2813/3309/2303/2076/1329/830/337/164/76/23/3/0/0/0
	Test	3653/9650/11277/11246/9579/6487/4269/3258/1860/1121/712/380/117/12/3	916/2467/2849/1935/1746/1137/782/304/160/60/20/1/0/0/0
	Valid	2501/6308/7302/7206/5945/3961/2625/2027/1135/746/449/203/77/8/1	513/1476/1703/1196/1040/627/419/167/76/39/13/0/1/0/0
Gasch2	Train	4626/12297/14105/14154/11855/8003/5171/4075/2348/1448/870/401/110/14/0	1088/2852/3357/2339/2103/1344/836/339/164/74/25/2/0/0/0
	Test	3632/9635/11344/11267/9501/6495/4277/3258/1847/1110/700/369/119/13/4	914/2503/2919/1976/1781/1153/790/308/160/56/20/1/0/0/0
	Valid	2475/6251/7269/7131/5928/3984/2663/2042/1147/765/459/204/79/8/1	525/1509/1721/1217/1054/637/420/160/72/38/17/0/1/0/0
Hom	Train	4647/12828/14387/14138/11919/8025/5190/4050/2302/1412/837/378/104/11/0	1059/2867/3305/2298/2072/1327/823/333/164/76/23/3/0/0/0
	Test	3732/10161/11654/11506/9831/6683/4412/3330/1848/1119/709/379/117/12/3	918/2521/2848/1925/1743/1131/782/305/159/60/20/1/0/0/0
	Valid	2538/6660/7547/7352/6095/4075/2705/2068/1133/745/449/203/77/8/1	511/1518/1705/1190/1037/626/418/167/76/39/13/0/1/0/0
Pheno	Train	1899/4896/5656/5751/4816/3216/2034/1609/930/598/383/183/51/6/0	430/1140/1349/969/844/551/345/121/58/26/8/2/0/0/0
	Test	1730/4487/5273/5248/4449/3030/2023/1593/921/536/334/181/55/5/1	443/1124/1354/934/802/526/401/152/78/34/14/1/0/0/0
	Valid	1033/2747/3193/3203/2693/1806/1198/937/549/344/223/111/50/7/1	236/650/786/546/474/267/202/84/35/18/7/0/1/0/0
Spo	Train	4503/11999/13716/13620/11436/7667/4959/3914/2261/1391/833/380/103/11/0	1042/2754/3256/2266/2038/1317/823/331/163/76/23/3/0/0/0
	Test	3636/9581/11192/11149/9504/6440/4249/3228/1849/1115/710/379/117/12/3	908/2436/2829/1918/1721/1126/773/302/160/60/20/1/0/0/0
	Valid	2469/6244/7250/7158/5917/3944/2612/2018/1135/746/449/203/77/8/1	511/1469/1691/1187/1033/623/417/167/76/39/13/0/1/0/0
Seq	Train	4731/13009/14603/14357/12098/8146/5254/4106/2328/1425/841/383/104/11/0	1079/2922/3360/2343/2123/1352/833/339/165/75/23/3/0/0/0
	Test	3788/10282/11809/11644/9939/6748/4447/3376/1878/1129/713/380/117/12/3	932/2561/2895/1959/1778/1148/790/306/160/60/20/1/0/0/0
	Valid	2564/6698/7588/7398/6127/4100/2720/2074/1135/745/449/204/78/8/1	518/1536/1723/1207/1054/640/426/169/77/40/13/0/1/0/0
Struc	Train	4664/12533/14259/14102/11846/7955/5136/4040/2324/1421/838/382/104/11/0	1077/2857/3354/2339/2119/1350/831/339/165/75/23/3/0/0/0
	Test	3686/9858/11465/11390/9628/6519/4292/3285/1850/1113/704/380/121/12/3	930/2511/2883/1958/1773/1132/790/305/156/57/19/1/0/0/0
	Valid	2529/6456/7412/7275/6006/4010/2663/2046/1135/745/449/204/78/8/1	517/1501/1717/1201/1052/637/425/169/76/40/13/0/1/0/0
Mean	Train	4224/11356/12968/12899/10819/7270/4689/3705/2133/1317/789/360/97/10/0	985/2613/3062/2133/1923/1241/773/310/151/69/21/2/0/0/0
	Test	3378/9033/10559/10501/8911/6049/3986/3050/1734/1043/659/351/109/11/3	851/2307/2660/1803/1622/1053/732/284/149/55/18/0/0/0/0
	Valid	2289/5884/6794/6689/5542/3703/2455/1897/1062/699/422/193/75/7/1	479/1386/1581/1109/968/583/392/155/70/36/13/0/1/0/0

As shown in Table 7, there was a similar behaviour as in the FunCat update. There was a substantial increase in the number of labels throughout all levels, specially in the levels between the third and the twelfth. Two extra levels were added, making a total of 15, nonetheless there are only few classes in these levels.

We observed an overall increase in the number of instances per level throughout the hierarchies (Table 8). There were no remarkable decreases. We have noticed that only the validation and test datasets contain instances on the last level of the hierarchy. From the machine learning perspective, such condition might hinder predictive models, as most of them are not capable of predicting a class which is not present in the training dataset. Possibly, future studies might consider removing the last level. Difficulties might also emerge on the fourteenth level, as the datasets have very few instances on it.

As seen in Table 9, once again there was an increment in the number of annotations per level. The number of annotations gradually increases up to a certain level, until it decreases to almost none when it reaches the deepest levels.

When examining the number of annotations that are added or removed per level (Table 10), we can perceive once again an overall increment in all datasets. Naturally, no labels were removed on the fourteenth and fifteenth level as they were not present in the 2007 versions.

## Results

Initially, we present a standard evaluation among the HMC methods. Next, we also present an alternative evaluation where the HMC methods are compared w.r.t. their ability to discover new or wrong annotations.

### Standard evaluation

In Table 11, we present a comparison of the PooledAUPRC obtained using the standard evaluation procedure. Since HMC-LMLP, HMC-GA and AWX are stochastic, we report the mean result of 5 runs, together with the standard deviation. Mind that, since we reran all methods on our datasets, variations may occur compared to the originally reported results in the respective papers.

**Table 11:** Pooled AUPRC of the evaluated methods

	Method	Cellcycle	Derisi	Expr	Eisen	Gasch1	Gasch2	Seq	Spo	Struc	Hom
FunCat 2007	Clus-Ensemble	0.222	0.187	0.257	0.268	0.258	0.226	0.257	0.211	0.198	0.293
	HMC-GA	0.131 ±0.002	0.111 ±0.012	0.145 ±0.027	0.141 ±0.018	0.146 ±0.011	0.135 ±0.01	0.142 ±0.03	0.135 ±0.01	-	-
	HMC-LMLP	0.110 ±0.004	0.107 ±0.002	0.104 ±0.007	0.110 ±0.003	0.105 ±0.002	0.108 ±0.003	0.105 ±0.004	0.107 ±0.002	-	-
	AWX	0.119 ±0.001	0.032 ±0.004	0.023 ±0.003	0.161 ±0.002	0.162 ±0.001	0.125 ±0.001	0.03 ±0.002	0.032 ±0.005	0.118 ±0.005	0.09 ±0.007
FunCat 2018	Clus-Ensemble	0.356	0.361	0.410	0.380	0.411	0.392	0.414	0.370	0.225	0.363
	HMC-GA	0.128 ±0.013	0.120 ±0.014	0.165 ±0.013	0.153 ±0.009	0.134 ±0.013	0.129 ±0.012	0.128 ±0.017	0.115 ±0.011	-	-
	HMC-LMLP	0.188 ±0.003	0.167 ±0.002	0.219 ±0.003	0.241 ±0.002	0.216 ±0.005	0.194 ±0.004	0.228 ±0.002	0.173 ±0.000	-	-
	AWX	0.137 ±0.002	0.034 ±0.004	0.026 ±0.004	0.185 ±0.001	0.178 ±0.002	0.148 ±0.001	0.032 ±0.01	0.036 ±0.002	0.145 ±0.017	0.06 ±0.006
Gene Ontology 2007	Clus-Ensemble	0.236	0.205	0.266	0.284	0.264	0.243	0.282	0.230	0.372	0.431
	HMC-GA	0.304 ±0.015	0.250 ±0.042	0.335 ±0.018	0.317 ±0.026	0.317 ±0.016	0.324 ±0.011	0.311 ±0.044	0.273 ±0.024	-	-
	HMC-LMLP	0.334 ±0.002	0.334 ±0.002	0.343 ±0.003	0.376 ±0.003	0.348 ±0.005	0.343 ±0.001	0.340 ±0.003	0.313 ±0.002	-	-
	AWX	0.205 ±0.028	0.048 ±0.001	0.033 ±0.017	0.200 ±0.026	0.160 ±0.002	0.240 ±0.018	0.060 ±0.007	0.052 ±0.009	0.261 ±0.006	0.122 ±0.052
Gene Ontology 2018	Clus-Ensemble	0.384	0.368	0.406	0.425	0.409	0.391	0.437	0.374	0.348	0.429
	HMC-GA	0.296 ±0.013	0.273 ±0.043	0.326 ±0.017	0.328 ±0.025	0.307 ±0.031	0.310 ±0.018	0.338 ±0.016	0.298 ±0.002	-	-
	HMC-LMLP	0.349 ±0.000	0.339 ±0.000	0.357 ±0.000	0.395 ±0.000	0.365 ±0.000	0.361 ±0.000	0.375 ±0.000	0.337 ±0.000	-	-
	AWX	0.204 ±0.028	0.066 ±0.003	0.026 ±0.009	0.197 ±0.026	0.161 ±0.015	0.235 ±0.01	0.071 ±0.01	0.059 ±0.006	0.164 ±0.023	0.039 ±0.002

**Fig. 6:**
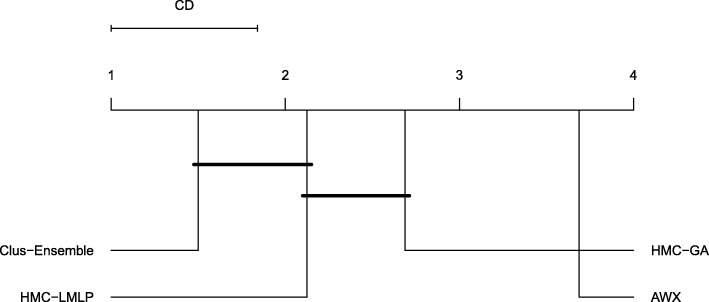
Friedmann-Nemenyi test evaluating the four HMC methods using the standard evaluation procedure

Even though Clus-Ensemble is the oldest of the compared methods, it still provided better results in most of the experiments. This is best seen in the FunCat 2018 datasets where Clus-Ensemble consistently presented results close to 0.4, and the second best method, HMC-LMLP, achieves at most 0.24 in any of the datasets. As can be seen in Fig. 6, Clus-Ensemble was the overall best method, and performs statistically significantly better than HMC-GA and AWX.

The second method evaluated, HMC-GA, yielded a lower performance overall. In most of the cases, HMC-GA was superior to AWX, but still inferior to Clus and HMC-LMLP. The method HMC-LMLP provided decent results. When compared to AWX, HMC-LMLP managed to significantly outperform it. Furthermore, HMC-LMLP was ranked as the second best method overall, providing superior results in all of the Gene Ontology 2007 datasets.

An unusual behaviour was noticed in the AWX method as it yielded very undesired results in many occasions. Even though the parameter values were extracted from the original paper, its results were fairly different. For instance, in the Derisi, Seq and Spo datasets from all versions, AWX was severely underfitted with results inferior to 0.1. It also presented similar cases in the FunCat and Gene Ontology 2007 Expr datasets.

When comparing the performance between different versions of the datasets, we noticed an overall improvement in the methods when moving from 2007 to 2018. Even though their label sets are bigger now, the addition of annotations to the instances compensate such difference, which resulted in better performances.

### vs 2018

Here we evaluate how the HMC methods perform when trained using data from 2007, but evaluated using datasets from 2018. For the methods HMC-LMLP, HMC-GA and AWX, for each (instance,label) pair we have used the mean prediction probability of 5 runs.

For all figures presented here, we also include a boxplot for the (instance,label) pairs that did not change between the two dataset versions. This allows to see to what extent the methods can detect annotations that were falsely negative or falsely positive in the data of 2007. The number between parentheses corresponds to the number of (instance,label) pairs evaluated for a particular setting and dataset. Note that the number of unchanged pairs is much higher than the number of changed pairs, hence the outliers (prediction probabilities outside the whisker) should not be regarded.

Furthermore, we have also employed the Friedman-Nemenyi test to provide statistical validation. In this case, we have used the difference between the median of the prediction probabilities for the annotations that changed and those that did not change between the two dataset versions.

#### FunCat

**Fig. 7:**
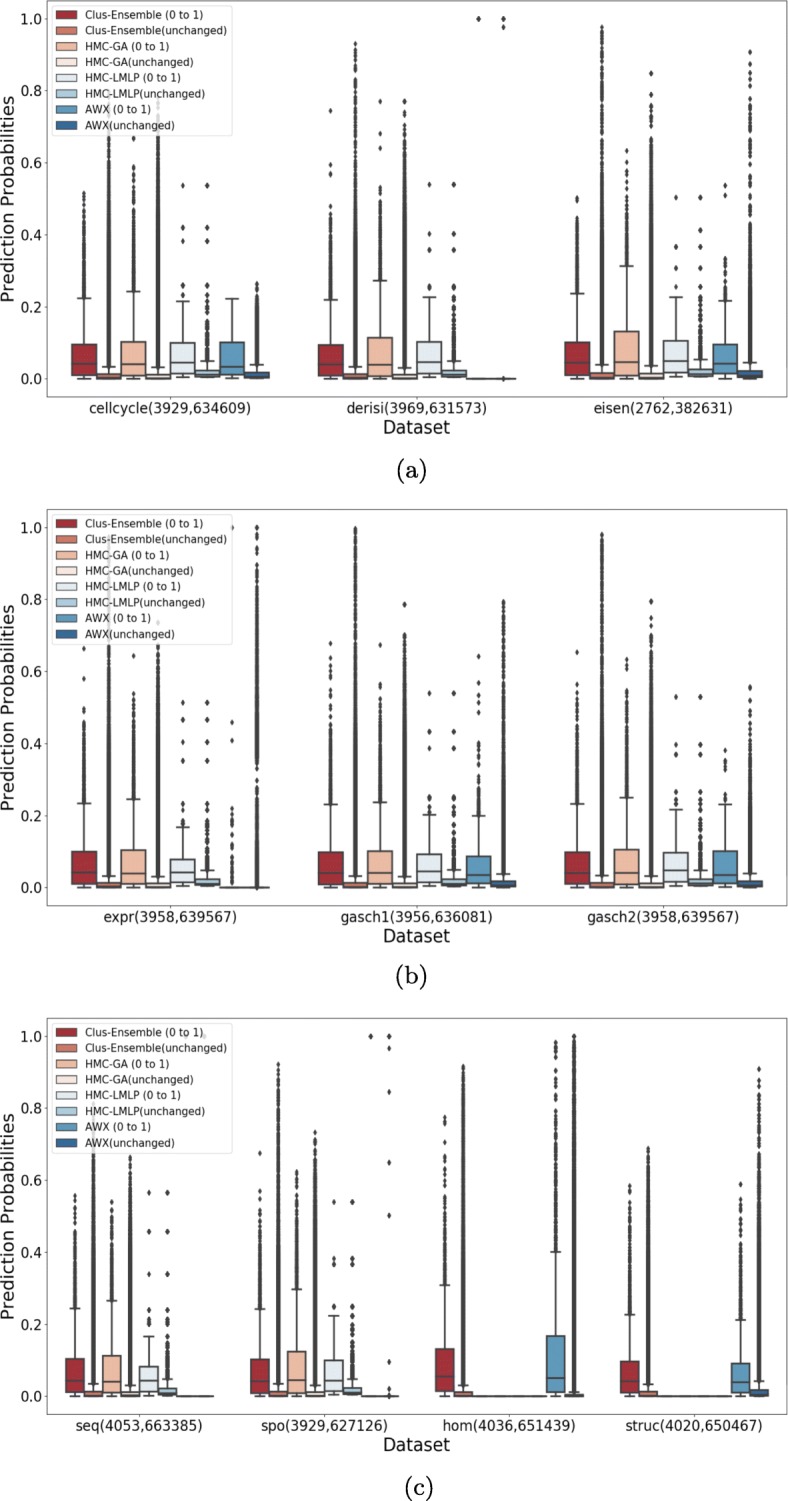
Evaluation on annotations that were added (0 to 1) and on annotations that did not change (0 in both versions) for FunCat. **a** Cellcycle, Derisi and Eisen datasets. **b** Expr, Gasch1 and Gasch 2 datasets. **c** Seq, Spo, Hom and Struc datasets

Figure 7 demonstrates that all methods are capable to detect missing annotations from the FunCat taxonomy, i.e., the distribution of prediction probabilities for the changed annotations is consistently higher than for the annotations that remained negative, since there is a visible difference between the location (median) and spread in the boxplots of the changed and unchanged annotations of the evaluated methods.

**Fig. 8:**
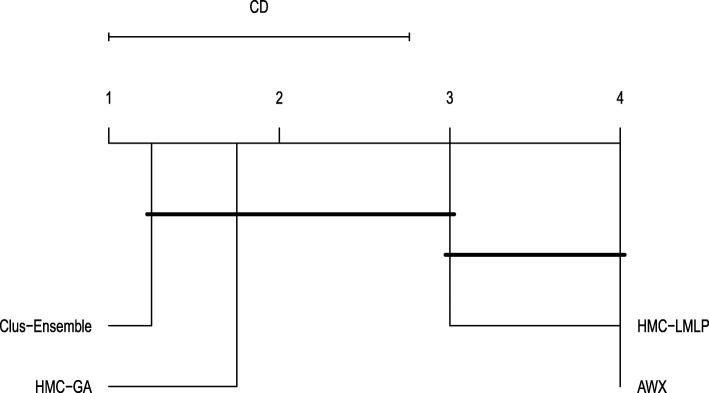
Friedman-Nemenyi test evaluating annotations that were added (FunCat)

Clus-Ensemble and HMC-GA provided similar results, however Clus-Ensemble was slightly superior since its prediction probabilities tended to be higher. Moreover, when evaluating the labels that did not change (remained absent), Clus-Ensemble provided very low prediction probabilities. In Fig. 8, Clus-Ensemble was ranked first, however not statistically different from HMC-GA and HMC-LMLP.

Similarly, the AWX method managed to be superior in the Hom dataset. However, it underperformed in other datasets, specially in Derisi, Expr, Seq and Spo. In these datasets, AWX predicted almost all annotations to be absent, except for very few outliers, which received a very high prediction probability.

HMC-LMLP presented decent results in almost all datasets. Nonetheless, for labels that did not change, HMC-LMLP tended to provide higher prediction probabilities, whereas Clus-Ensemble yielded lower ones, giving Clus-Ensemble an advantage over HMC-LMLP.

Hence, in the context of discovering new annotations, we can assume that Clus-Ensemble is the safer choice as it performed better on almost all datasets, nonetheless its advantage was close to minimal.

**Fig. 9:**
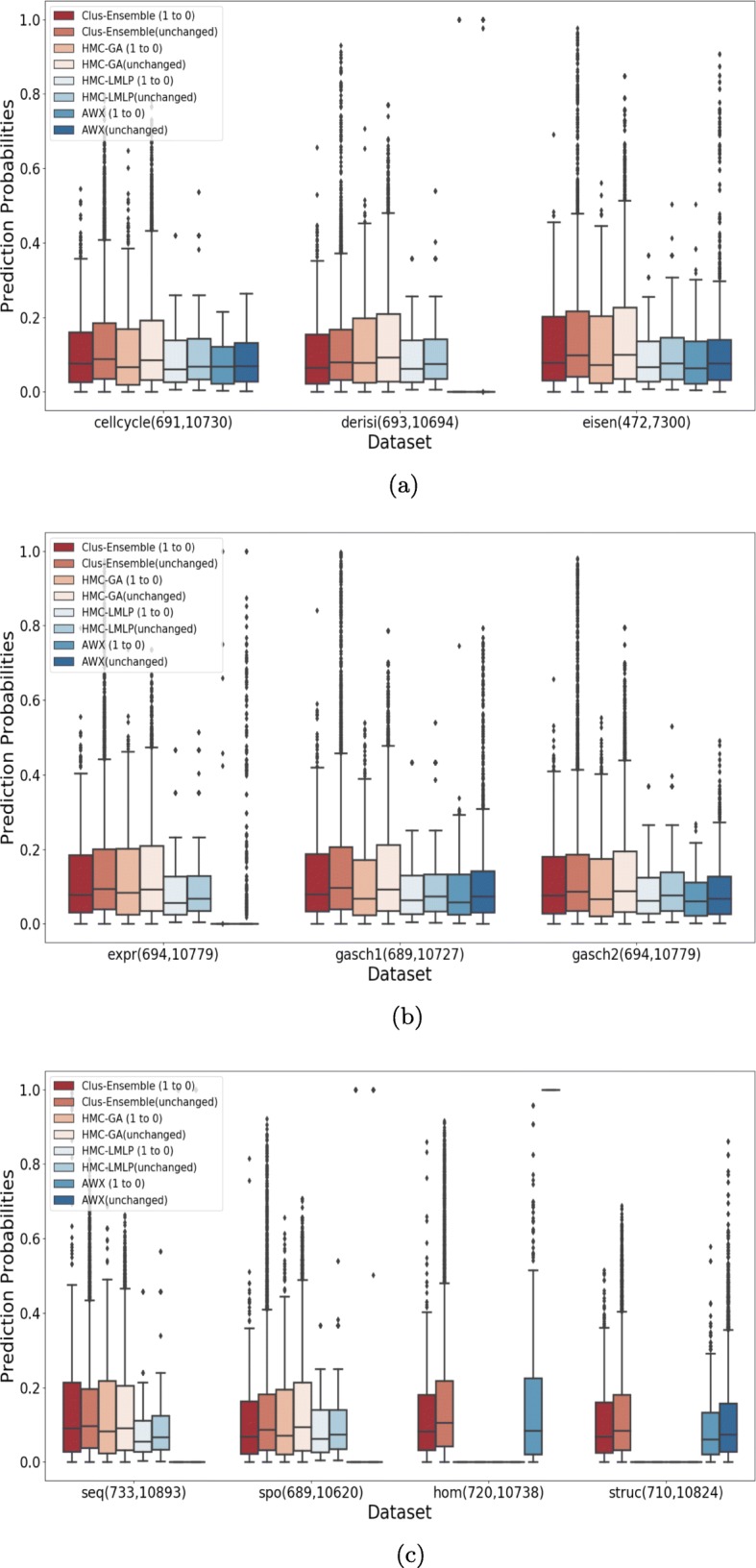
Evaluation on annotations that were removed (1 to 0) and on annotations that did not change (1 in both versions) for FunCat. **a** Cellcycle, Derisi and Eisen datasets. **b** Expr, Gasch1 and Gasch2 datasets. **c** Seq, Spo, Hom and Struc datasets

**Fig. 10:**
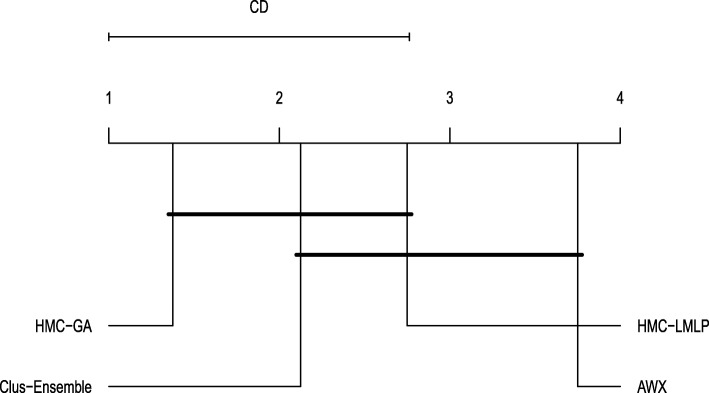
Friedman-Nemenyi test evaluating annotations that were removed (FunCat)

When addressing labels that were removed, see Fig. 9, we had very similar results. As seen in Fig. 10, HMC-GA provided superior results, but it still was not statistically different from Clus-Ensemble and HMC-LMLP. AWX yielded lower prediction probabilities in most of the datasets with exception to the Hom dataset. Since its prediction probabilities were also low for labels that were present in both versions of the datasets, it performs the worst among the compared methods.

#### Gene ontology

**Fig. 11:**
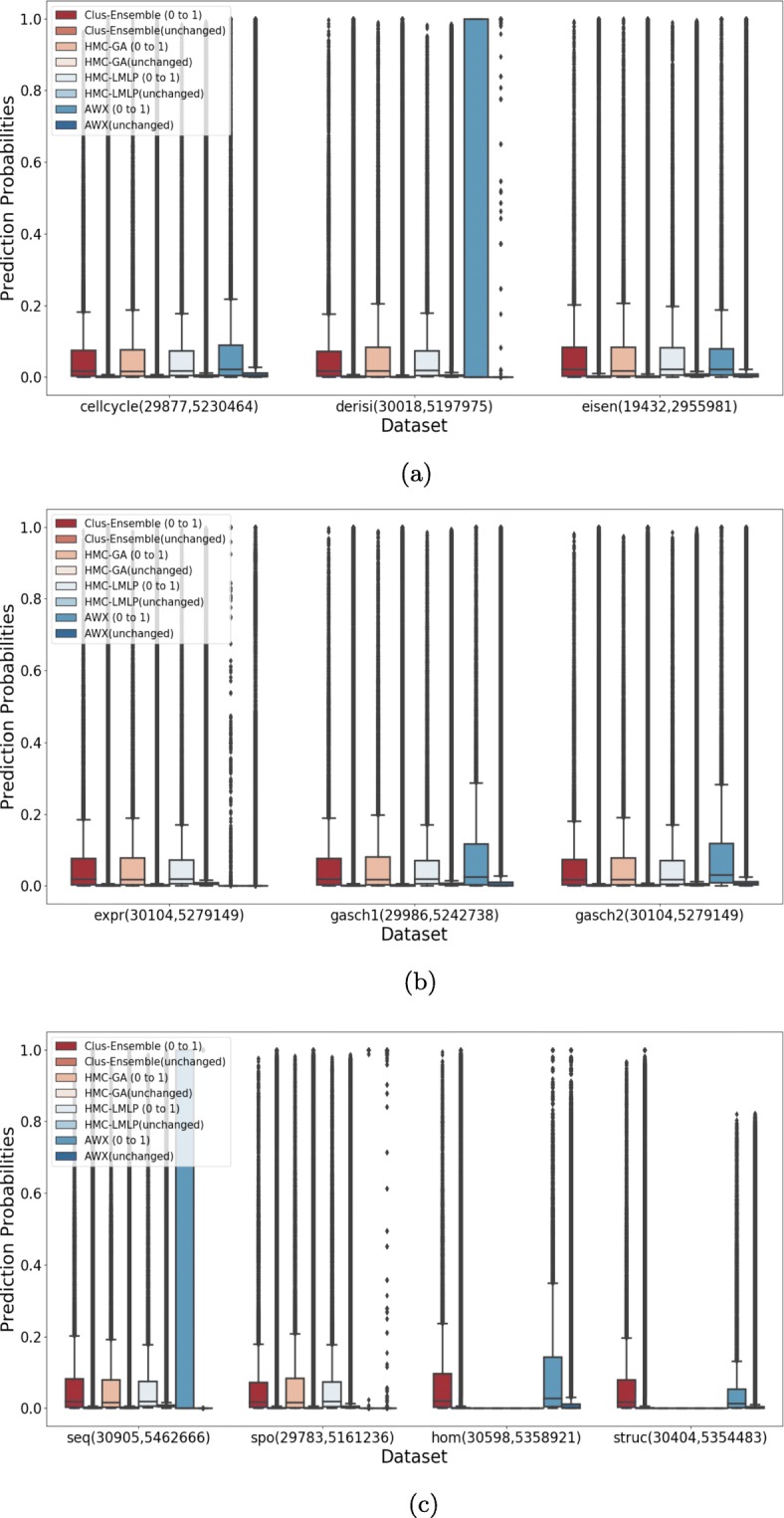
Evaluation on annotations that were added (0 to 1) and on annotations that did not change (0 in both versions) for GO. **a** Cellcycle, Derisi and Eisen datasets. **b** Expr, Gasch1 and Gasch2 datasets. **c** Seq, Spo, Hom and Struc datasets

**Fig. 12:**
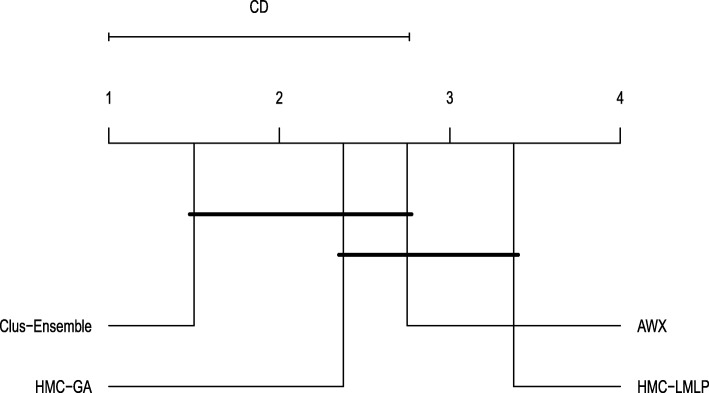
Friedman-Nemenyi test evaluating annotations that were added (GO)

As can be seen in Fig. 11, Clus-Ensemble and HMC-GA were superior in most of the datasets. Additionally, the AWX method also presented desirable results, specially in the Derisi and Seq datasets where it output very high probabilities for added annotations and very low ones for labels that did not change. These three methods were not statistically different from each other, as shown in Fig. 12.

The HMC-LMLP method also presented overall visually comparable results, nonetheless it yielded higher predictions for annotations that did not change in some datasets, such as Expr, Gasch1 and Gasch2.

**Fig. 13:**
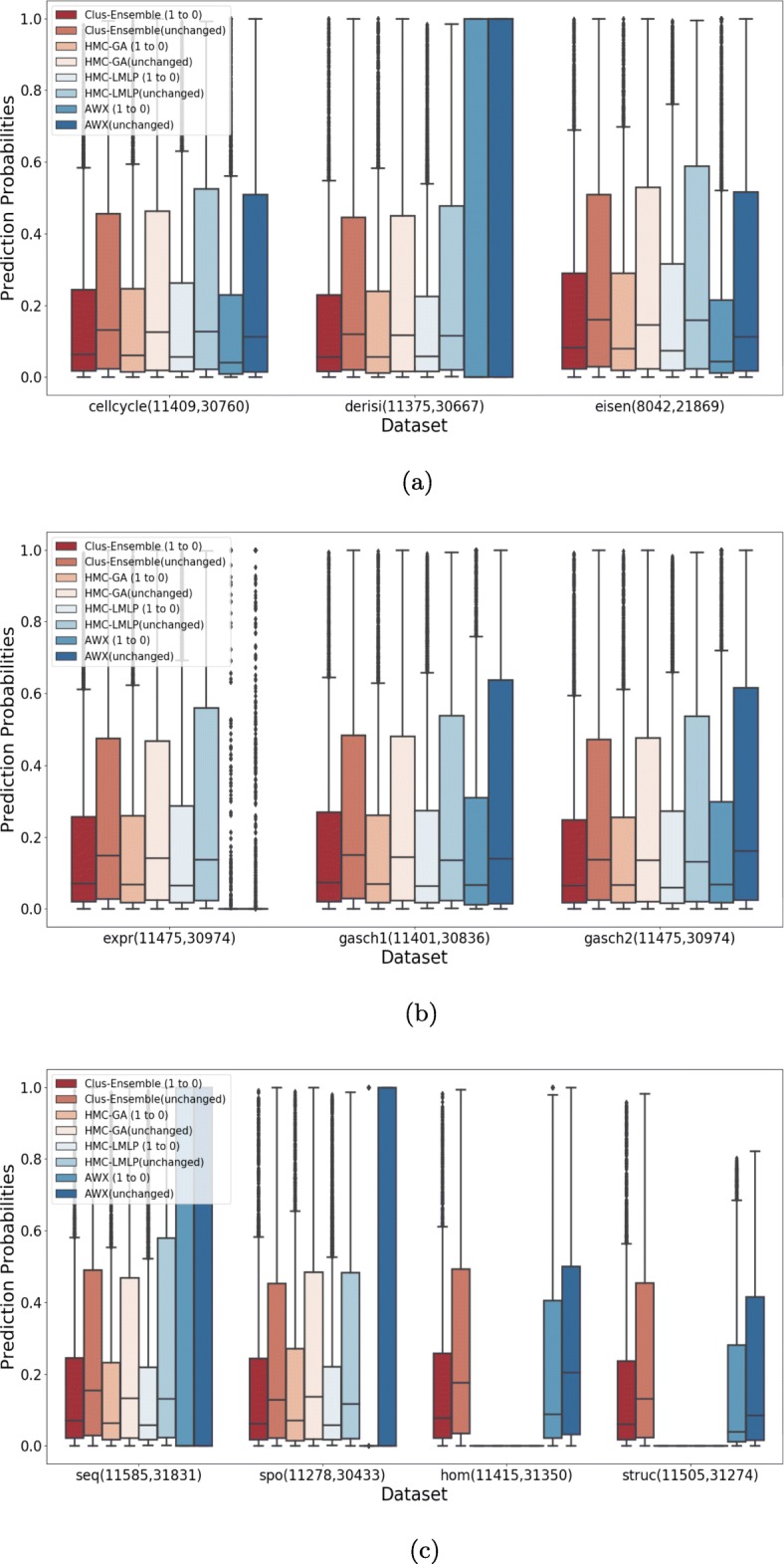
Evaluation on annotations that were removed (1 to 0) and on annotations that did not change (1 in both versions) for GO. **a** Cellcycle, Derisi and Eisen datasets. **b** Expr, Gasch1 and Gasch2 datasets. **c** Seq, Spo, Hom and Struc datasets

**Fig. 14:**
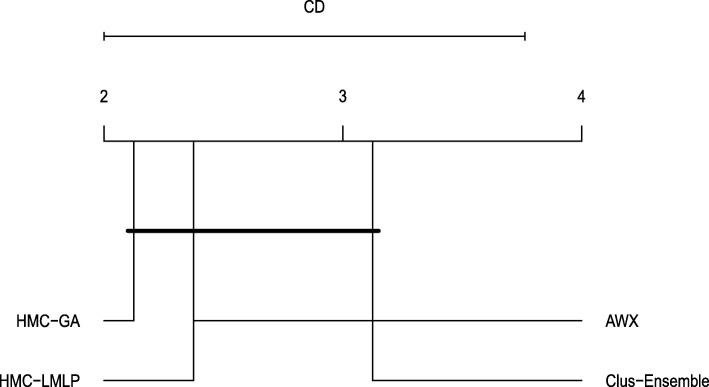
Friedman-Nemenyi test evaluating annotations that were removed (GO)

When examining the labels that were removed in Fig. 13, we noticed a different outcome. In this case, all methods presented very similar results, making performance almost indistinguishable in most of the datasets. Additionally, there was no statistical difference among these methods, as shown in Fig. 14.

## Discussion

In this section, we present a discussion about the results presented in the previous section. Following the same order, we first address the standard evaluation, followed by the comparison between the versions of the datasets.

### Standard evaluation

As shown in Fig. 6, Clus-Ensemble’s superior predictive performance, in combination with an efficient learning method (random forest), the ability to handle datasets with many features (as seen in the Struc and Hom datasets), and the interpretabilty aspect (e.g. variable ranking and proximity measure associated to random forests), confirm the state-of-the-art status of Clus-Ensemble.

We believe that the ensemble method, random forest, contributes substantially to the performance. By considering many models, Clus-Ensemble is able to generalize more, and consequently provide superior results. The other methods evaluated do not make use of any ensemble method. Even though HMC-LMLP contains many neural networks, they are trained as a single model, and they distinguish between different classes.

HMC-GA provided inferior results in many cases, nonetheless it has the highest interpretability since it generates classification rules. Similarly, Clus-Ensemble presents many trees, which are readable by themselves, however their interpretability decreases as the number of trees increases. Differently, the neural networks, HMC-LMLP and AWX, are black-box models, and thus not readable in a straightforward way.

When comparing the neural network methods, HMC-LMLP and AWX, HMC-LMLP clearly had the upper hand. We believe that this is due to HMC-LMLP being a local approach, whereas AWX is a global one. Since one neural network is trained for each level of the hierarchy, the neural networks are trained to distinguish among fewer classes, making the classification task easier, and, thus, providing better results. The computational complexity of HMC-LMLP, however, is considerably higher than the other methods due to many neural networks being built during its training.

Despite some undesirable results, AWX is the only method that explicitly exploits the hierarchy constraint by propagating gradients from neurons associated to leaves to neurons associated to their parents. Mind that the other methods also respect the constraint, but they exploit it to a smaller extent during their training.

Moreover, we believe that AWX’s early stopping criterion has negatively affected the results. in order to prevent overfitting, AWX interrupts the training right after the performance in the validation set decreases. However, these datasets contain noise in their label set, thus a small oscillation might be noticed. Considering more iterations, as performed by HMC-LMLP, could possibly increase AWX’s performance. Moreover, neural networks are very parameter dependent, and despite using the recommended parameters for all methods on the version from 2007, their performance might increase if they are tuned again on the 2018 datasets.

### vs 2018

#### FunCat

As described previously, when analyzing labels that changed from absent to present (0 to 1), Clus-Ensemble had the overall best results, whereas HMC-GA was the best for present to absent (1 to 0). We believe that this finding is highly correlated to how the evaluated methods yield their prediction probabilities.

Clus-Ensemble outputs the mean prediction probability of the instances associated to the leaf node predicted. According to the parameters used, the minimum number of such instances is 5, making the lowest positive prediction probability to be 0.2 per tree. Even though fairly low, it still is reasonably high in HMC due to label sparsity, resulting in high prediction probabilities in many cases, and thus better performance.

Likewise, the HMC-GA method yielded high prediction probabilities in some cases, resulting in similar results to Clus. Moreover, their heuristic (variance reduction) is the same. The main difference between HMC-GA and Clus-GA relies on the fact that HMC-GA uses a mean rule (prediction of the mean label set of the training dataset) whenever a test instance is not classified by any of the rules. This possibly results in outputting a sparse prediction with very low prediction probabilities.

Despite having decent results, HMC-LMLP presented high very prediction probabilities for labels that did not change between versions. We believe that this is related to how neural networks learn the distribution of the data. Since neural networks are very powerful models, they can learn more complex boundaries when compared to Clus-Ensemble and HMC-GA, resulting in the neural networks adjusting themselves strictly to the training dataset. HMC-LMLP is not overfitted though, as shown in Table 11, nonetheless its usage is not recommended if label noise is likely to be present.

Lastly, AWX had the best performance in the Hom dataset. However, it underperformed in several other cases. Once again, the early stopping criterion might have forced the neural network to a sub-optimal configuration, resulting in very biased predictions, i.e. AWX assumes most of the labels to be either positive or negative.

When evaluating labels that were removed, HMC-GA was superior. We believe that the mean rule might have artificially contributed since very low probabilities are predicted for most of the labels in this case.

#### Gene ontology

In the GO datasets, we noticed a similar behaviour. In most of the situations, Clus-Ensemble performed better when evaluating labels that were added, whereas HMC-GA was superior for removed labels.

When it comes to removed labels, HMC-GA performed better. Consequently, we recommend the usage of HMC-GA to predict which annotations are likely to be removed in future versions of the datasets (noise) since it presented better results in both FunCat and GO.

Similarly to the FunCat experiments, HMC-LMLP had an average performance being statistically significantly inferior to other methods, but equivalent to them for removed labels.

When compared to its performance on FunCat, AWX performed better here. For labels that were added, even though ranked in lower positions, AWX managed to not be statistically significantly different from Clus-Ensemble and Clus-HMC. Likewise, for removed labels, AWX also performed reasonably. This is very surprising since GO datasets have even more labels to be distinguished, and the same parameters were used.

## Conclusion

In this work, we have presented updated benchmark datasets for hierarchical multi-Label classification (HMC) in the area of protein function prediction. We have also performed a comparison among four HMC methods to provide baselines results on these datasets. Finally, we have proposed an alternative evaluation procedure to evaluate the ability of HMC methods to detect missing or wrong annotations. For this purpose we make use of both old and new versions of the datasets.

In all datasets, we have noticed a significant increase in the hierarchy size, and in the number of annotations associated to instances. As a consequence of that, when performing a standard evaluation, HMC methods performed better using the updated versions. Despite having more labels to distinguish, the instances have now more annotations associated to them, resulting in better predictions. The overall best method in this task was Clus-Ensemble, a random forest of decision trees adapted to HMC, nonetheless the results remained fairly low overall. Thus, protein function prediction is still a very challenging task for the machine learning community.

In this direction, further studies in this area are necessary. In particular, we instigate the use of Deep Learning methods, since the amount of data available is on a constant increase, and recent deep neural networks are capable of learning straight from DNA sequences (without the need of extracting features) [46].

When it comes to detect missing or wrong annotations, in the FunCat datasets, Clus-Ensemble was the best in detecting missing annotations, whereas HMC-GA did better for annotations that were removed. In the Gene Ontology datasets, Clus-Ensemble performed better for detecting missing annotations, and competitive results were obtained for wrong annotations.

To conclude, we recommend to use the updated datasets in future studies on this topic. However, the previous version of these datasets should not be disregarded, since having two versions can be of interest to perform an evaluation similar to ours on new HMC methods, or to other fields in machine learning such as weakly supervised classification, noise detection and incremental learning [47, 48].

## Methods

In this section, we provide details about our experimental setup. First, we present the methods used for comparison. Then we describe two evaluation strategies. Finally, we explain which datasets were included in the evaluation.

### Compared methods

We have compared 4 methods from the literature: Clus-Ensemble [2, 21], hierarchical multi-label classification with genetic algorithm (HMC-GA) [4, 19], hierarchical multi-label classification with local multi-layer perceptrons (HMC-LMLP) [3], and Adjacency Wrapping matriX (AWX) [6]. The methods were chosen due to the following reasons: 1) Apart from Clus-Ensemble, they are recent methods. Clus-Ensemble is included because it is used as the state-of-art benchmark in many studies; 2) They are based on different machine learning methods and HMC strategies, ranging from global to local approaches and from interpretable tree or rule based methods to more powerful, but black box techniques; 3) They are publicly available. Next, we provide a brief description of these methods, and details about their parameters. We have set the parameters to the values originally recommended by the authors.

#### Clus-Ensemble

Clus is a method from the global approach based on predictive clustering trees where decision trees are seen as a hierarchy of clusters whose top node corresponds to a cluster with all the training data. Recursively, Clus minimizes the intra-cluster variance until a stop criterion is met. In this work, we have used the (global) Clus-HMC variant due to its superior results, in combination with the ensemble method Random Forest. Hence, this predictive model consists of a Random Forest of Predictive Clustering Trees. We are using 50 trees within the Random Forest, at least 5 instances per leaf node and the best F-test stopping criterion significance level selected from {0.001,0.005,0.01,0.05,0.1,0.125}.

#### HMC-GA

Using genetic algorithms and the global approach, the method hierarchical multi-label classification with genetic algorithm use a sequential rule covering method where optimal classification rules are created [4, 19]. At every iteration, one rule in the format *if*→*then* is generated by optimizing the fitness function. Next, the examples covered by the new rule are removed from the training dataset, and new rules are generated until a stop criterion is met. We have used the following parameters:

- **Population size** : 100 rules;
- **Number of Generations**: 1000;
- **Stopping criterion**: 1% of uncovered examples;
- **Crossover rate**: 90%;
- **Mutation rate**: 10%;

#### HMC-LMLP

The method proposed by Cerri [3] addresses the classification problem using the Local approach. More specifically, the Local Classifier per Level strategy where one multi-layer perceptron is trained for each level of the hierarchy. Thus, each neural network is responsible for predicting the classes on its respective level. Moreover, this method adds prediction probabilities from the previous level as extra features for the next neural network, in the sense that each neural network is trained separately and its training dataset is augmented by the previous neural network. Finally, the predictions from each neural network are combined to perform a prediction. If the performance in the validation dataset does not improve in 10 iterations, the training is interrupted.

We have used the following parameters:

- **Hidden Layers Size**: the number of neurons per hidden layer is obtained by multiplying the number of inputs by the values [0.6,0.5,0.4,0.3,0.2,0.1] for the FunCat datasets and [0.65,0.65,0.6,0.55,0.5,0.45,0.4,0.35,0.3,0.25,0.2,0.15,0.1] for the GO datasets;
- **Activation Function**: Logistic (sigmoid) activation function;
- **Optimizer**: Backpropagation with 200 epochs and learning rate ∈{0.05,0.03} and momentum ∈{0.03,0.01} alternating between levels;

#### AWX

Using neural networks and the global approach, the method Adjacency Wrapping matriX (AWX) employs a single model where the underlying hierarchy is mapped into the loss function [6]. This mapping is performed by an auxiliary matrix which makes the gradients updates flow from the neurons associated to leaves to the neurons that are associated to their parent nodes. If the performance degrades on the validation dataset, the training is interrupted immediately. We have used the following parameters:

- **l-norm**: We have used *l*_1_, since it presented superior results;
- **Hidden layer**: with 1000 neurons with the ReLu activation function and *l*_2_ regularizer 10^−3^;
- **Output layer**: Logistic activation function and *l*_2_ regularizer 10^−3^;
- **Optimizer**: Adam with learning rate 10^−5^,*β*_1_=0.9 and *β*_2_=0.999 and the cross entropy loss function;

### Evaluated datasets

Even though we provide 12 datasets with updated Funcat and GO annotations, we have decided to not include all of them in our analysis. The Church and Pheno datasets have an unusual number of instances with identical feature vectors, mostly due to missing values. In the Church dataset, 2352 out of 3755 instances are unique, leaving 1403 instances with the same feature vector as another instances, but different annotations. A similar behaviour is noticed in the Pheno dataset where only 514 instances out of 1591 are unique [49].

We are considering the Hom and Struc datasets only using the methods Clus-Ensemble and AWX. The other methods, HMC-LMLP and HMC-GA, presented several difficulties when handling these datasets. HMC-LMLP demands much more computational power due to its many neural networks. Similarly, HMC-GA did not converge using the parameters suggested in the original paper. Some work, such as [5, 10, 11, 13, 17, 22], have also decided to not include them.

Table 12 presents the datasets evaluated in this work.

**Table 12:** Evaluated datasets

Dataset	#Features	#Instances	#Funcat2007	#FunCat2018	#GO2007	#GO2018
Cellcycle	77	3757	499	585	4122	8065
Derisi	63	3725	499	585	4116	8039
Eisen	79	2424	461	552	3570	6993
Expr	551	3779	499	585	4128	8067
Gasch1	173	3764	499	585	4122	8064
Gasch2	52	3779	499	585	4128	8067
Seq	478	3919	455	586	3124	8075
Spo	80	3703	499	585	4130	8040
Struc	19628	3851	499	586	4132	8070
Hom	47034	3867	499	585	4126	8054

### Standard evaluation

In order to provide benchmark results on the new datasets, we have first performed a standard evaluation. Thus, we evaluated 10 feature sets with 4 possible labels sets for each (two label hierarchies and two annotation timestamps), making a total of 40 datasets. We present the evaluation measure and the statistical test that we have used.

#### Pooled aUPRC

We have adopted the Pooled area under the precision-recall curve (AUPRC) evaluation measure since it is consistently used in HMC literature [2, 3, 5, 18, 19, 21, 22, 25]. Mind that, generally HMC datasets are heavily imbalanced, making negative predictions very likely, thus evaluation measures such as ROC curves are not recommended.

The Pooled AUPRC corresponds to the area under the precision-recall curve generated by taking the Pooled (i.e., micro-averaged) precision and recall over all classes for different threshold values. These threshold values usually consist of values ranging from 0 to 1 with increasing steps of 0.02 for all datasets.

In the equations below, *tp* stands for true positive, *fp* means false positive, *fn* refers to false negative and *i* ranges over all classes.

1 $$  Pooled\_precision = \frac{\sum{tp_{i}}}{\sum{tp_{i}} + \sum{fp_{i}}}  $$

2 $$  Pooled\_recall = \frac{\sum{tp_{i}}}{\sum{tp_{i}} + \sum{fn_{i}}}  $$

#### Friedman-Nemenyi test

In order to provide statistical evidence, we have used the Friedman-Nemenyi test. At first the Friedman test verifies if any of the compared methods performs statistically significantly different from others. Next, the Nemenyi test ranks the methods where methods with superior results are ranked in higher positions. Graphically, methods connected by a horizontal bar of length equal to a critical distance are not statistically significantly different.

### Evaluation procedure to compare datasets from different versions

We also investigated whether models that were trained on a dataset from 2007 are able to discover new annotations, i.e., annotations that were unknown (negative) in 2007, but have been added afterwards. We also check the opposite situation: whether models are able to correct wrong annotations, i.e., annotations that were wrongly positive in 2007, and have been corrected to negative afterwards. For this purpose, we propose an evaluation strategy that compares the predicted probabilities for specific (instance,label) pairs over the different HMC methods.

**Fig. 15:**
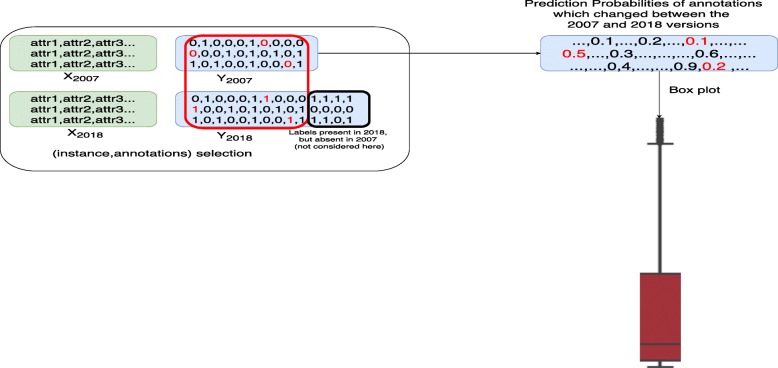
Prediction probabilities of labels that changed between versions (written in red inside the red box) are used to build the red box-plot. Labels that occur only in the 2018 versions are not considered in this evaluation (black box)

In particular, for a fair comparison, first we take the intersection of the label sets in the 2007 and 2018 dataset versions, respectively. Then, for evaluating the discovery of new annotations, in this intersection, we check the (instance,label) pairs in the test set that were negative in 2007 and positive in 2018. For these pairs, we plot the distribution of predictions for each HMC method, trained on the 2007 dataset. Note that a high value would have yielded a false positive prediction in 2007, however, with the current knowledge in functional genomics, this would now yield a true positive prediction. Figure 15 illustrates the procedure. For evaluating the correction of wrong annotations, the procedure is similar, except that we look for positive pairs that became negative.

## Data Availability

The datasets from 2007 and the Clus-Ensemble method are available at https://dtai.cs.kuleuven.be/clus/. The methods HMC-GA and HMC-LMLP are available at http://www.biomal.ufscar.br/resources.html. The AWX method is available at https://github.com/lucamasera/AWX. The new dataset versions are available at: https://www.kuleuven-kulak.be/nl/onderzoek/itec/projects/research-focus/software.

## Abbreviations

AUPRC: Area under the precision-recall curve
AWX: Adjacency wrapping matriX
FunCat: Functional catalogue
GO: Gene ontology
HMC: Hierarchical multi-label classification
HMC-GA: Hierarchical multi-label classification with genetic algorithm
HMC-LMLP: Hierarchical multi-label classification with local multi-layer perceptrons
UniProt: Universal protein

## Footnotes

1 Available in: https://www.kuleuven-kulak.be/nl/onderzoek/itec/projects/researchfocus/software

2
https://blast.ncbi.nlm.nih.gov/Blast.cgi

3
https://www.uniprot.org/uniprot/
